# Dynamic models for musical rhythm perception and coordination

**DOI:** 10.3389/fncom.2023.1151895

**Published:** 2023-05-17

**Authors:** Edward W. Large, Iran Roman, Ji Chul Kim, Jonathan Cannon, Jesse K. Pazdera, Laurel J. Trainor, John Rinzel, Amitabha Bose

**Affiliations:** ^1^Department of Psychological Sciences, University of Connecticut, Mansfield, CT, United States; ^2^Department of Physics, University of Connecticut, Mansfield, CT, United States; ^3^Music and Audio Research Laboratory, New York University, New York, NY, United States; ^4^Department of Psychology, Neuroscience and Behaviour, McMaster University, Hamilton, ON, Canada; ^5^Center for Neural Science, New York University, New York, NY, United States; ^6^Courant Institute of Mathematical Sciences, New York University, New York, NY, United States; ^7^Department of Mathematical Sciences, New Jersey Institute of Technology, Newark, NJ, United States

**Keywords:** beat perception, entrainment, neuro-mechanistic modeling, dynamical systems, music, Bayesian modeling, synchronization

## Abstract

Rhythmicity permeates large parts of human experience. Humans generate various motor and brain rhythms spanning a range of frequencies. We also experience and synchronize to externally imposed rhythmicity, for example from music and song or from the 24-h light-dark cycles of the sun. In the context of music, humans have the ability to perceive, generate, and anticipate rhythmic structures, for example, “the beat.” Experimental and behavioral studies offer clues about the biophysical and neural mechanisms that underlie our rhythmic abilities, and about different brain areas that are involved but many open questions remain. In this paper, we review several theoretical and computational approaches, each centered at different levels of description, that address specific aspects of musical rhythmic generation, perception, attention, perception-action coordination, and learning. We survey methods and results from applications of dynamical systems theory, neuro-mechanistic modeling, and Bayesian inference. Some frameworks rely on synchronization of intrinsic brain rhythms that span the relevant frequency range; some formulations involve real-time adaptation schemes for error-correction to align the phase and frequency of a dedicated circuit; others involve learning and dynamically adjusting expectations to make rhythm tracking predictions. Each of the approaches, while initially designed to answer specific questions, offers the possibility of being integrated into a larger framework that provides insights into our ability to perceive and generate rhythmic patterns.

## 1. Introduction

Biological processes, actions, perceptions, thoughts, and emotions all unfold over time. Some types of sensory information like static images can carry meaning independent of time, but most, like music and language, get all of their semantic and emotive content from temporal and sequential structure. *Rhythm* (see **Glossary**) can be defined as the temporal arrangements of sensory stimuli offering some amount of temporal predictability. The predictability granted by rhythm allows the organism to prepare for and coordinate with future events at both neural and behavioral levels (deGuzman and Kelso, 1991; Large and Jones, 1999; Schubotz, 2007; Fujioka et al., 2012; Patel and Iversen, 2014; Vuust and Witek, 2014). For example, repetitive movements, such as locomotion, are rhythmic. Simple repetitive rhythmic movements are relatively easy to instantiate in neural circuits, as can be seen from work on invertebrates and central pattern generation circuits (Kopell and Ermentrout, 1988; Marder et al., 2005; Yuste et al., 2005). Rhythm is also the basic building block for human auditory communication systems, speech, and music (e.g., Fiveash et al., 2021), but here, of course, the dynamics are much more complex and flexible.

**FIGURE 1:**
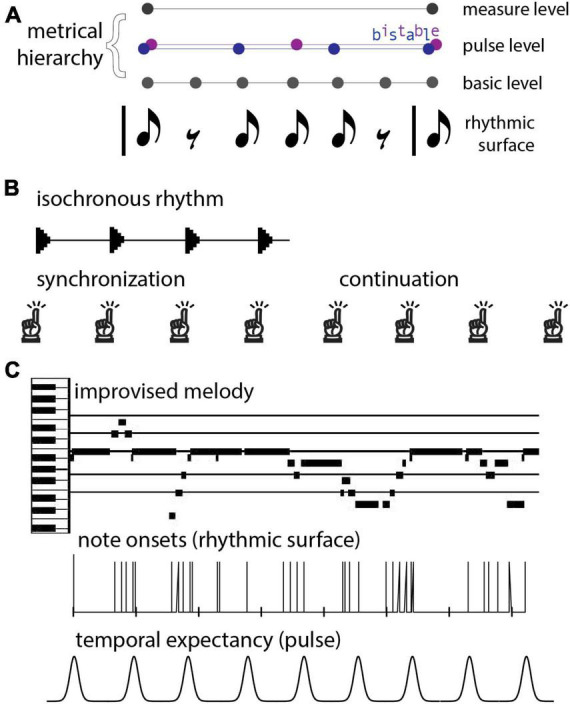
**(A)** A rhythmic pattern (rhythmic surface) and possible metrical hierarchies. Notes indicate sound onsets and rests indicate silences. People perceive beats at different rates, or levels, and people neurally extract periodic sequences corresponding to beats with events at sounds and silences. Three levels of the metrical hierarchy (beat levels) are shown. In the example, the pulse (the intermediate beat level) is bistable; people can perceive this rhythm in groups of 2 or in groups of 3 pulses. **(B)** The synchronization-continuation task. A person first listens to an isochronous rhythm and synchronizes movements with it. Next, the stimulus ceases and the person continues tapping at the same frequency. **(C)** An improvised piano melody. Musical events unfold with diverse timings and discrete onset times are not periodic, but may result in a pseudo-periodic pulse percept, which can function as a series of temporal expectancies (i.e., times at which events are likely to occur) in the human listener.

From a rhythmic pattern (*rhythmic surface*), humans neurally extract a (typically) periodic sequence known as a *beat.* It should be noted that beats can be perceived even where the rhythmic surface has a rest or silence (Figure 1A). Humans perceive beats at rates between about 0.5–8 Hz, with optimal beat perception around 2 Hz (Repp, 2005; Patel and Iversen, 2014). In addition, human brains extract a *metrical hierarchy* of temporal organization (or nested beat levels), typically with groups of two or three successive beat events at one level forming a single beat event at another level (see Figure 1A). The term *pulse* refers to the most salient level of beats, behaviorally defined as the beats a listener taps when synchronizing with a musical rhythm. Importantly, energy at the pulse frequency, or *tempo*, of a rhythm is not necessarily contained prominently, or not at all, in the rhythmic surface (Haegens and Zion Golumbic, 2018). Thus, the pulse percept comes from the brain’s tendency to impose rhythmic structure on its auditory input (Tal et al., 2017), which it begins to do even early in development (Phillips-Silver and Trainor, 2005). It should be noted also that for complex rhythms, the beat can be ambiguous or multi-stable; for example, a 6-beat pattern could be organized hierarchically as two groups of three beats or as three groups of two beats (see Figure 1A). In this review we focus on models of beat, pulse, and meter, aimed at comparing and contrasting theoretical and modeling frameworks. While the approaches we review have also been used to make predictions about other rhythmic phenomena such as pattern perception (Kaplan et al., 2022) and groove, these topics are beyond the scope of the current review.

The major developmental disorders, including dyslexia, autism, attention deficits, and developmental coordination disorder, have been associated with deficits in timing and rhythm processing (Lense et al., 2021), suggesting that rhythm perception is deeply intertwined with core developmental processes. Rhythm is an important ingredient of human social functioning: the predictability of auditory rhythms enables humans to plan movements so as to synchronize with the beat, which in turn facilitates synchrony between people. A number of studies have shown that interpersonal synchrony promotes feelings of trust and social bonding (Hove and Risen, 2009; Valdesolo et al., 2010; Mogan et al., 2017; Savage et al., 2020), even in infancy (Cirelli et al., 2014; Trainor and Marsh-Rollo, 2019). The centrality of rhythm to human development and social interaction might explain why rhythmic music is universal across human societies; why people engage in music (listening and/or playing) most days of their lives; why caregivers around the world use rhythmic infant-directed singing as a tool to help infants with emotional regulation (calming or rousing) and social development; and why adults engage in rhythmic music to promote social bonding, such as at weddings, funerals, parties, team sports, cultural rituals, in war, and in religious ceremonies.

The central role of rhythm perception in human life energizes the scientific challenge of understanding it. In the present paper, we examine how different classes of computational models approach the problem of how rhythms are perceived and coordinated, focusing on the strengths and challenges faced by each approach. In the remainder of the introduction, we outline some of the basic issues that models must address. Then, in subsequent sections, we go into details of the predominant approaches to date.

At the most basic level, humans are able to perceive pulse and meter, and synchronize their motor behavior with music, such as through dance (Savage et al., 2015; Ellamil et al., 2016), body sway (Burger et al., 2014; Chang et al., 2017), or finger tapping (Repp, 2005; Repp and Su, 2013). Even synchronizing with an *isochronous sequence* of events, such as that produced by a metronome, raises numerous theoretical questions. Does the brain develop an explicit model of the metronome to predict its timing, or do the brain’s intrinsic neural rhythms naturally entrain to the beat? Might the brain eschew the use of oscillatory processes, and instead estimate the passage of time by counting the pulses of a pacemaker? Or does it utilize a combination of endogenous oscillatory neuronal circuits and counting processes?

Human rhythmic capabilities are simultaneously flexible enough to adapt to changes in musical tempo, yet robust enough to maintain the beat when faced with perturbations. Research on synchronization suggests that people can re-synchronize their tapping within a few beats of a tempo change (Large et al., 2002; Repp and Keller, 2004; Loehr et al., 2011; Scheurich et al., 2020); therefore, any model of rhythm perception must be able to flexibly adapt in real-time, within several beats. Furthermore, music production often occurs as a collaborative activity, in which multiple performers must coordinate with one another in real time. Within these social contexts, the flexibility of human synchrony allows performers to maintain cohesion through mutual adaptation to one another’s variations in timing (Konvalinka et al., 2010; Loehr and Palmer, 2011; Dotov et al., 2022). How this adaptation occurs remains an open question: Do timing changes evoke prediction errors that drive the brain to update its model of the meter, or do perturbations directly drive neural oscillations to shift in phase and/or period? Rhythm perception is also robust to brief perturbations. In real performances, musicians use deviations from “perfect” mechanical timing as a communicative device. For example, musicians often leave clues as to the metrical structure of a piece by lengthening the intervals between notes at phrase boundaries (Sloboda, 1983; Todd, 1985), called phrase-final lengthening. Research suggests that these instances of expressive timing may actually aid – rather than disrupt – listeners in parsing the metrical and melodic structure of the music (Sloboda, 1983; Palmer, 1996; Large and Palmer, 2002). Alternatively, small timing deviations appear to be related to information content or entropy. Phrase boundaries are typically places of high uncertainty about what notes, chords, or rhythm will come next, and there is evidence that people slow down at points of high uncertainty (Hansen et al., 2021). A viable neural model of rhythm perception must therefore not destabilize or lose track of the beat due to intermittent deviations such as these.

Our sense of rhythm is robust not only to timing perturbations but also to the complete discontinuation of auditory input: humans display the ability to continue tapping to a beat even after an external acoustic stimulus has been removed (e.g., Chen et al., 2002; Zamm et al., 2018), known as *synchronization-continuation* tapping (Figure 1B). Does this ability imply that we have “learned” the tempo? Or perhaps it points to hysteresis in a neural limit cycle, allowing it to maintain a rhythm even after external driving has ceased.

Important for its theoretical implications is the fact that synchronization behavior appears to be anticipatory. For example, when synchronizing finger taps with rhythms, people tend to tap tens of milliseconds before the beat – a phenomenon known as *negative mean asynchrony* (NMA; see Repp and Su, 2013, for a review). What neural mechanisms might be responsible for this asynchrony? Does NMA represent a prediction of an upcoming event, or might it arise from a time-delayed feedback system among neural oscillators (e.g., Dubois, 2001; Stepp and Turvey, 2010; Roman et al., 2019)?

Beyond the basic beat structure, music can be far more intricate and involves perceiving, learning, and processing complex acoustical patterns, such as in the case of *syncopation*. Evidence suggests that people can still perceive a beat in highly complex rhythms (see Figure 1C), even rhythms so complex as to contain no spectral content at the beat frequency (Large et al., 2015; Tal et al., 2017). This ability necessitates considering larger network structures that appropriately respond to the different frequencies present in these patterns. What kinds of neural structures might be at play? Perhaps they are oscillator-based, and if so, the extent to which the intrinsic and synaptic properties of the constituent elements determine model output is important to understand. Perhaps these networks form a rhythm pattern generator akin to a “central pattern generator” found in motor systems, cardiac systems, and various invertebrate systems (Kopell and Ermentrout, 1988; Marder et al., 2005; Yuste et al., 2005) in which case it would be important to understand what is the neural basis for pacemaking within the circuit. On the other hand, it is possible that the neural structures do not rely on intrinsic oscillation at all. For example, there may exist stored templates used to perceive and recall a rhythm. If so, it is of interest to explore plausible neural instantiations for template learning or other possible non-oscillatory mechanisms.

Electrophysiological data from non-human primates has gradually shed light on some of the possible mechanisms of beat and rhythm processing. One main challenge is the time (years) and effort that goes into training monkeys to synchronize with isochronous beats and complex rhythmic stimuli. Nonetheless, data from non-human primates has revealed that individual neurons in the medial prefrontal cortex (mPFC) show accelerating firing rates that reset after beat onsets and are a direct function of the beat tempo (Crowe et al., 2014; Merchant and Averbeck, 2017; Zhou et al., 2020). These studies reveal that the mPFC dynamically encodes the beat, and that it can integrate periodic information independent of the stimulus modality (i.e., visual or auditory; Betancourt et al., 2022). To date we are not aware of human data collected at comparable neural substrates during a similar rhythm-tracking task.

In humans it is extremely difficult to measure single neuron activity, hampering the development of detailed biophysical models of neurons and the circuits that underlie human rhythm processing. However, higher level models can be informed by non-invasive methods in humans. In particular scalp-measured EEG and MEG have the temporal resolution to observe both population neural oscillatory activity in the time-frequency domain as well as large brain events occurring over tens of milliseconds. fMRI recordings are not sensitive enough to observe fine temporal dynamics, but they are able to delineate brain areas involved in rhythmic processing, as well as the connections between these areas. Despite these limitations, human neurophysiological data has informed some higher-level models of rhythm processing, both in suggesting constraints and providing a way to test some aspects of the model predictions. In summary, computational modeling efforts attempt to address fundamental questions about how the brain generates a beat and maintains it in the presence of noise and perturbations, while retaining the flexibility to adapt on the time scale of seconds to new tempos or rhythms, whether they are simple like that of a metronome (Figure 1B) or more complex like that found in a piece of music (Figure 1C).

In this paper, we review multiple modeling paradigms at three different levels of description that seek to explain various aspects of human rhythmic capabilities. These models rely, to some extent, on the fact that the organized temporal structure of musical rhythms is generally predictable and often periodic. At the highest level of organization, Bayesian inference (e.g., Sadakata et al., 2006; Cannon, 2021) and predictive coding (e.g., Vuust and Witek, 2014; Koelsch et al., 2019) approaches exploit the predictability of rhythms by suggesting that the brain constructs a statistical model of the meter and uses it to anticipate the progression of a song. In contrast, oscillator models based in the theory of dynamical systems utilize Neural Resonance Theory (Large and Snyder, 2009) to show how structure inherent in the rhythm itself allows anticipatory behavior to emerge through the coupling of neural oscillations with musical stimuli (e.g., Large and Palmer, 2002; Large et al., 2015; Tichko and Large, 2019). In this approach, a heterogeneous oscillator network spanning the frequency range of interest mode-locks to complex musical rhythms At perhaps the most basic level of modeling, beat perception of isochronous rhythms is addressed by mechanistic models that are either event-based (Mates, 1994a,b; Repp, 2001a; van der Steen and Keller, 2013) or based on neuronal-level oscillator descriptions (Bose et al., 2019; Byrne et al., 2020; Egger et al., 2020), both of which fall within the broad framework of dynamical systems. These mechanistic models postulate the existence of error-correction processes that adjust the period and phase of the perceived beat. Evaluating the merits of each modeling approach requires testing how well each can address specific findings from among the rich collection of rhythmic abilities and tendencies that humans exhibit.

The topic of musical rhythm has a rich history in psychology, and many different types of models have been proposed (Essens and Povel, 1985; McAngus Todd and Brown, 1996; Cariani, 2002). The models reviewed in this paper share the common feature that they are all dynamic; namely, they all involve variables that evolve in time according to some underlying set of ordinary differential equations. Another common feature is event-based updating; an event may be the onset of an auditory stimulus, the spike time of a neuron, or the zero crossing of an oscillator’s phase. At each event time, a variable or a parameter in the model is updated with a new value. In some cases, this may lead to formulation as a discrete dynamical system that can be described using a mathematical map (e.g., Large and Kolen, 1994; Large and Jones, 1999). Despite these formal similarities, the way that the dynamic formalisms are deployed often reflects deep differences in theoretical approaches. The neuro-mechanistic approach utilizes differential equations that describe the dynamics of individual neurons, which may then be combined into circuits designed to implement descriptions of neural function. The neuron-level and often the circuit-level descriptions can be analyzed using the tools of nonlinear dynamical systems (Rinzel and Ermentrout, 1998; Izhikevich, 2007), and the resulting models are well-suited to making predictions about neuron-level data. The dynamical systems approach is a theoretical framework within which the embodied view of cognition is often formalized (Kelso, 1995; Large, 2008; Schöner, 2008). Here, differential equations capture the general properties of families of physiological dynamical systems, and are derived using the tools of nonlinear dynamics (Pikovsky et al., 2001; Strogatz, 2015). These models are not intended to make predictions about neuron-level data. Rather, they are aimed at making predictions about ecological dynamics (dynamics of interaction of the organism with its environment), population-level neural dynamics, and the relationship between the two. Finally, Bayesian inference models are not always associated with dynamics, however the domain of musical rhythm (which is all about change in time) makes the language of differential equations natural in this case. Bayesian models such as the one described here are aimed squarely at behavior-level data, not at the level of individual neurons or even neural populations. However, when deployed in the rhythmic domain, interesting parallels emerge that relate Bayesian models to more physiologically oriented descriptions, as we shall see.

## 2. Neuroscience of rhythm processing

There is considerable evidence that the brain is capable of both duration-based (or single interval) timing and beat-based timing (Kasdan et al., 2022). fMRI and TMS studies in healthy populations show that duration-based timing relies particularly on the cerebellum (Teki et al., 2011; Merchant et al., 2015), and these results are corroborated in patients with cerebellar damage (Grahn and Brett, 2009; Grube et al., 2010). Furthermore, the cerebellum has been associated with prediction, absolute duration, and error detection (Kasdan et al., 2022). On the other hand, fMRI studies consistently show that beat perception activates a network involving auditory superior temporal regions, basal ganglia (notably the putamen), and supplementary (and pre-supplementary) motor areas (Lewis et al., 2004; Chen et al., 2006, 2008a,b; Grahn and Brett, 2007; Bengtsson et al., 2009; Grahn and Rowe, 2009, 2013; Teki et al., 2012; Patel and Iversen, 2014). It has been hypothesized that beat perception relies on the integration of these systems through pathways additionally involving parietal and auditory areas (Patel and Iversen, 2014; Cannon and Patel, 2021). Critical to this hypothesis is that beat perception relies on auditory-motor interactions. In humans, there is much evidence that motor areas are used for auditory timing (Schubotz, 2007; Iversen et al., 2009; Fujioka et al., 2012). Evidence from non-human primates synchronizing with periodic stimuli also shows interactions between sensory and motor areas (Betancourt et al., 2022). Thus, fully modeling rhythm processing in the human brain will likely require an architecture with both local areas and bidirectional interactions between areas.

Cross-species studies suggest that humans and monkeys share mechanisms for duration-based timing, but that only humans show robust beat-based timing (Merchant and Honing, 2014; Merchant et al., 2015). Developmental studies indicate that young infants detect changes in rhythms in simple as well as moderately complex metrical structures, but that by 12 months of age, infants have already become specialized for processing rhythms with metrical structures that are common in their environment (Hannon and Trainor, 2007). Cross-cultural perceptual/tapping studies in adults have revealed that there is a universal tendency for people to “regularize” randomly timed rhythm patterns, so as to perceive/reproduce small-integer ratios between the durational intervals (Jacoby and McDermott, 2017). At the same time, people who grew up in different cultures that exposed them to different meters show different biases for the ratios that dominate their perception and production (Jacoby et al., 2021). Thus, of relevance to modeling, there appear to be both innate tendencies that may reflect intrinsic properties of neural networks and a degree of flexibility, such that networks are likely shaped both by the immediate input and longer-term learning.

Many neuroscience studies of beat and rhythm have been theoretically motivated by the idea that temporal regularity enables prediction of when upcoming beats are expected (Large and Kolen, 1994; Huron, 2008), with the consequence of enhanced perception at the times of beat onsets in determining both when events are expected to occur and what the events are expected to be (Large and Jones, 1999; Schroeder and Lakatos, 2009; Arnal and Giraud, 2012; Henry and Herrmann, 2014; Chang et al., 2019). While fMRI studies can reveal brain regions involved in processing rhythmic information, the temporal and spectral precision of EEG and MEG make them useful for testing models of rhythm perception. Two basic approaches can be taken for EEG analysis. One examines the time course of electrical activity in the brain as sound events occur in a stimulus input (event-related potentials or ERPs), whereas the other uses frequency-based analyses to understand periodicities and phase relations in the neural response. Components of ERPs, such as mismatch negativity (MMN), occur in response to unexpected events in sequences or patterns of sounds (Näätänen et al., 2007; Carbajal and Malmierca, 2018), and have been shown to reflect predictive processes (e.g., Bendixen et al., 2009; Dauer et al., 2020). While clear MMN is seen in response to a sound event with unexpected pitch or timbre (or pattern of pitch or timbre events) in the context of a sequence of sound events, direct evidence for predictive timing is less clear. This is likely because it is difficult to distinguish neural ERP activity at the expected time of an event (representing a response to the violation of timing expectation) from neural activity in response to the actual sound event, whether it occurs earlier or later than expected. However, ERP responses have been shown to be larger for sound omissions on metrically strong beats than omissions on metrically weak beats (Bouwer et al., 2014), even in newborns (Winkler et al., 2009). ERPs can be used indirectly to examine temporal expectations, however. For example, infants show larger ERP responses on beats they were previously primed to perceive as accented compared to those they were primed to hear as unaccented, when listening to rhythms with ambiguous metrical structure (Flaten et al., 2022). Thus, even infants can endogenously maintain a particular metrical interpretation of an ambiguous rhythm, and models of rhythm perception need to be able to cope with ambiguity and multi-stability.

A popular approach has been to examine the entrainment of neural responses to the frequencies present in temporally structured auditory input stimuli, with an interest both in frequency and phase alignment (Henry et al., 2014; Morillon and Schroeder, 2015). Many studies show that when presented with a rhythm, whether in speech or music, ongoing delta frequencies will phase align with the beats of the rhythm (Schroeder and Lakatos, 2009; Lakatos et al., 2013, 2016; Poeppel and Assaneo, 2020). There is debate as to whether these neural oscillations represent the brain entraining to the input (Haegens and Zion Golumbic, 2018). Evidence suggests, however, that these oscillations do not simply reflect ERP responses following each sound event in the input stimulus (Doelling et al., 2019), but rather change according to predictive cues in the input (Herbst and Obleser, 2019). Further, neural oscillations do not simply mimic the temporal structure of the input (Nozaradan et al., 2011, 2012; Tal et al., 2017), but also reflect internally driven (endogenously activated) processes, some of which appear to be present already in premature infants (Edalati et al., 2023). Thus, neural oscillations appear to be a window into dynamic neural mechanisms (Doelling and Assaneo, 2021) and provide a way to test aspects of computational models.

The next few sections will examine and compare different modeling approaches spanning neuronal, mid-level and high-level descriptions of rhythm processing.

## 3. Models for time-keeping, beat generation, and beat perception

Models that allow for discrete event-times are suited to address questions related to time-keeping, generation of isochronous rhythms, and synchronization of a periodic rhythm to a complex musical rhythm (beat perception). Time-keeper models are error correction models within the information processing tradition, and they primarily operate by adapting to the time intervals in a stimulus. Continuous time neuro-mechanistic models described in this section produce event-times. They are also error-correction models that seek to align the timing of internally generated events to the timing of the external sound onsets. They update parameters of a dynamical neuronal system to achieve matching of phase and period with the stimulus. Adaptive oscillator models simulate synchronization (i.e., phase-and mode-locking) of an oscillation with a complex stimulus rhythm, and have been used to simulate the perception of a beat in complex musical rhythms. They adapt period based on the phase of individual events rather than on the measurement of time-intervals.

### 3.1. Algorithmic time-keeper models

The earliest set of time-keeper models were predominantly algorithmic error-correction models (Michon, 1967; Hary and Moore, 1987; Mates, 1994a,b; Vorberg and Wing, 1996), many of which were reviewed by Repp (2005) and Repp and Su (2013). For example, Mates (1994b) defined variables that model the internal time estimates that an individual makes; the *k*th stimulus onset time is defined as *S*_*I(k)*_, the *k*th motor response time by *R*_*I(k)*_, their error *e*_*I*(*k*)_ = *S*_*I*(*k*)_ - *R*_*I*(*k*)_ and the *k*th cycle period by *t*_*I(k)*_. At the occurrence of the stimulus, the period is updated by the correction rule


$$t_{I}\,\left(k\right)=t_{I}\,\left(k-1\right)-\beta \,\left(t_{I}\,\left(k-1\right)-\left[S_{I}\,\left(k\right)-S_{I}\,\left(k-1\right)\right]\right),$$

while phase correction obeys


$$R_{I}\,\left(k+1\right)=R_{I}\,\left(k\right)+t_{I}\,\left(k\right)-\alpha \,e_{I}\,\left(k\right),$$

where α and β are the strengths of the phase and period correction, respectively. This is a linear model which can be solved explicitly. In the isochronous case, *S*_*I*_(*k*) − *S*_*I*_(*k* − 1) equals the interonset interval, denoted *IOI* (the time between successive stimulus spikes) provided that the estimate is itself error free. In that case, period matching requires *t*_*I*_(*k* − 1) = *S*_*I*_(*k*) − *S*_*I*_(*k* − 1), which in turn implies *t*_*I*_(*k*) = *t*_*I*_(*k* − 1). Perfect synchrony for this case occurs when *e*_*I*_(*k*) = 0, *t*_*I*_(*k*) = *IOI* and thus the *R*_*I*_(*k* + 1) motor response is exactly an *IOI* in duration after the *R*_*I*_(*k*) motor response. Because the model is linear, it is straightforward to understand the separate effects that phase correction (strength α) and period correction (strength β) have on solutions. Further, the model readily adapts to tempo changes over a few cycles by changing *S*_*I*_(*k*) − *S*_*I*_(*k* − 1) away from a constant value. It can also exhibit asynchronies in the steady state difference between stimulus time and motor response by allowing *e*_*I*_(*k*) to be non-zero.

### 3.2. Neuro-mechanistic oscillator models

While time-keeper models have been successful in matching experimental data and aspects of synchronization, they leave open a major question. What are the neuro-mechanisms that the brain uses to synchronize and adapt to time intervals? Does the brain measure the passage of time? Early models focused on determining the length of intervals, anywhere from seconds to minutes. Treisman (1963), Church and Gibbon (1982), and Gibbon et al. (1984) proposed a pacemaker accumulator framework in which counts of a pacemaker clock are accumulated in a reference memory after a series of trials of different duration. These reference durations are then compared to a working memory accumulation for the current interval to make a judgment of duration. Left open is how a neuronal model may produce the pacemaker clock or the ability to count cycles and compare different counts. Also, an unaddressed issue is that in rhythmic interval timing, the model seeks a particular alignment of stimulus and beat generator phase; in single interval timing the accumulator is by definition aligned with the interval’s initial time.

**FIGURE 2:**
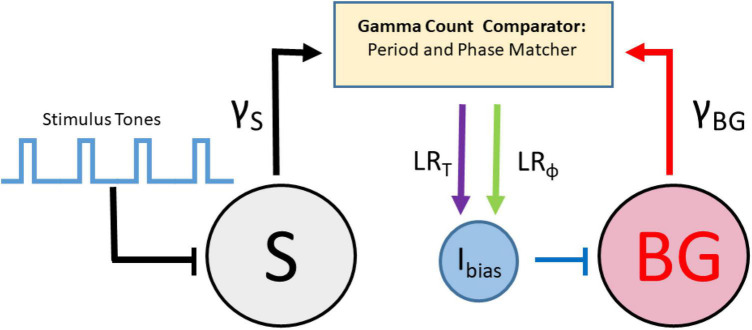
Schematic representation of the beat generator model with stimulus tones that drive a stimulus neuron (S), a beat generator neuron (BG) driven by an adjustable applied current (I_bias_), a gamma count comparator that changes I_bias_ through rules for period learning LR_T_ and phase learning LR_ϕ_.

**FIGURE 3:**
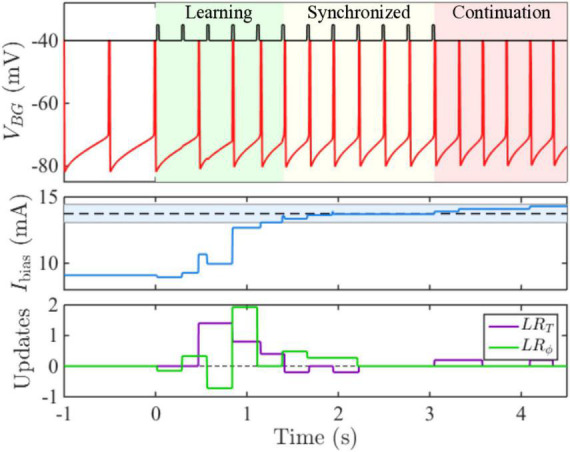
Time courses show the BG originally oscillating at 2 Hz quickly learning a faster 3.65 Hz frequency and then performing synchronization-continuation. **(middle panel)** Shows how I_bias_ adjusts within a few cycles based on period and phase learning rules **(lower panel)** to bring its value within a specified tolerance of the target value (dashed line). The blue shaded region represents the accuracy tolerance of roughly 30 ms prescribed in the model.

Recently, a new set of models for beat perception that are based on continuous time dynamical systems which incorporate event-based error correction rules have been derived. Bose et al. (2019) combined ideas of counting taken from pacemaker-accumulator models with those from error-correction models to develop an adaptive, biophysically based neuron/population beat generator (BG) that learns the spiking phase and period of a stimulus neuron (S) that represents an isochronous stimulus tone sequence (Figure 2). While based in dynamical systems, it is neither an information processing model, nor an entrainment model. It consists of a limit-cycle oscillator with specific period and phase learning rules that adaptively adjust an input strength I_*bias*_ to the BG. The term I_*bias*_ in conjunction with the widely found ionic currents that constitute the BG enabling it to oscillate over frequencies 0.5–8.0 Hz, that include beyond the range covered in finger-tapping experiments; (see Repp, 2005), determines the neuron’s frequency to input (freq-Input) curve. The BG neuron is a Type I neuron (oscillations can arise with arbitrarily slow frequencies as the input parameter increases through a bifurcation value) with a monotonic, non-linear freq-input curve whose shape determines several of the BG’s dynamic properties (Bose et al., 2019). It is not an entrainment model, as S does not exert an explicit forcing input to the BG. It differs from an information processing model by having an explicit neuronal representation of the beat generator, rather than positing the existence of an internal timekeeper. Indeed, as opposed to earlier algorithmic models, the error-correction rules are not directly applied to period and phase. Instead, the model compares estimates of IOI lengths by tracking the number of pacemaker cycles (e.g., gamma-frequency oscillations) between two successive BG spikes, γ_*BG*_, and two successive S spikes, γ_*S*_ through a gamma count comparator. A period correction learning rule *LR*_*T*_ is then applied at each BG firing event that adjusts *I_bias_* → *I*_*bias*_ + δ_*T*_(γ_*BG*_ - γ_*S*_) that tries to match the integer values of γ_*BG*_ to γ_*S*_. A phase correction learning rule LR_ϕ_ is implemented at each S firing event to adjust *I*_*bias*_ → *I*_*bias*_ + δ_*ϕ*_ *q*(*ϕ*)ϕ|1 − *ϕ*| and is designed to send *ϕ* to either 0 or 1. This rule has some hidden asymmetries and may contribute to the existence of NMA that the BG neuron displays. Here ϕ is defined as the number of gamma cycles from a BG spike to the next S spike divided by γ_*S*_, and *q*(*ϕ*) = *sgn*(*ϕ* − 0.5). The model resynchronizes quickly over a few cycles to changes in stimulus tempo (Figure 3) or phase, as well as to deviant or distracting stimulus events. It can perform synchronization-continuation (Figure 3) and displays NMA in that, on average, the BG firing time precedes that of the stimulus. Byrne et al. (2020) analyzed dynamical systems features of a BG model based on the integrate and fire neuron in computational and mathematical detail. Zemlianova et al. (2022) provided a biophysically based Wilson-Cowan description of a linear array network that propagates forward a single active excitatory-inhibitory (E-I) pair with each gamma cycle, thereby representing the current gamma count to estimate interval duration.

Egger et al. (2020) derived a related continuous time dynamical systems based error-correction model. They use a firing rate framework to describe the activity of neuronal populations in a feedforward two-layer (sensory and motor command) architecture that learns an isochronous stimulus sequence. Each layer incorporates an effective ramping variable, one for sensory and another for motor, that evolves toward a threshold. The ramping variable is derived from a competition dynamics that represents approximately the motion (time scale in IOI range) along the unstable manifold away from a saddle toward the steady state of dominance for one competitor. The ramping speed depends upon an input parameter *I* that drives the competition. Its value is adjusted at each reset in the sensory layer toward a match with the IOI of the stimulus. The motor layer (with similar competition dynamics) has a continuous-in-time adjustment of *I* to achieve phase alignment. The model accounts for a change in tempo as seen in their behavioral experiments. It also exhibits features of Bayesian performance in short 1–2-go and 1–3-go sequence timing tasks. In these tasks, participants are presented either 2 or 3 isochronous tones from a prior distribution and then asked to estimate the next beat. An optimal Bayesian integrator would utilize prior knowledge which would produce an estimate that is biased toward the mean of the distribution, which the model is able to reproduce. The authors observed a modest NMA in simulations, but the effect was not analyzed, and no attempt was made to fit empirical data. Future incorporation of time delays may however produce this effect (Ciszak et al., 2004; Nasuto and Hayashi, 2019; Roman et al., 2019; see also section “6.2.1. Transmission delay and negative mean asynchrony”).

### 3.3. Phase oscillator models and dynamic attending theory (DAT)

Another important early approach involved phase oscillator models (Large and Kolen, 1994; McAuley, 1995; Large and Jones, 1999; Large and Palmer, 2002) that entrain to their input. Theoretically, these oscillator models are based on the idea that the brain does not measure time, instead, the model maps time onto the phase of an oscillator that synchronizes to rhythmic events. Dynamic Attending Theory (DAT) refers to the hypothesis that the neural mechanisms of attention are intrinsically oscillatory, and can be entrained by external stimuli, allowing attention to be directed toward specific points in time (Jones, 1976; Jones and Boltz, 1989; Large and Jones, 1999).

#### 3.3.1. Circle map phase oscillators models

**FIGURE 4:**
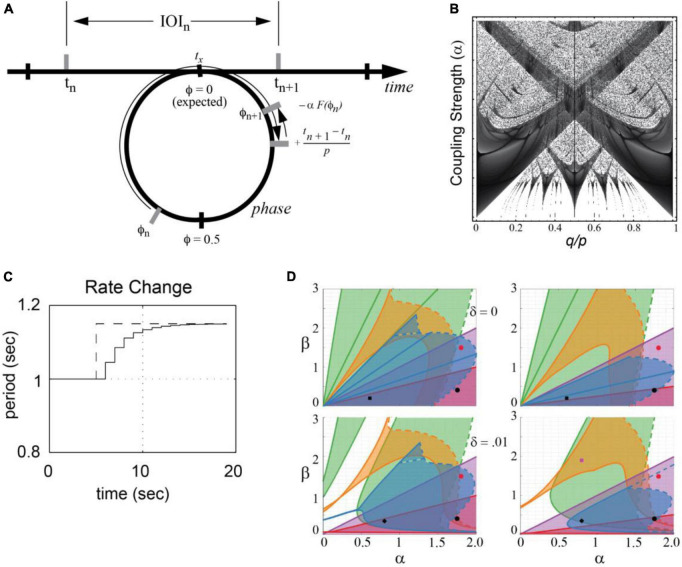
**(A)** A circle map maps events at times *t_n_* and *t*_*n–1*_ onto the phase of an oscillator that entrains to the event sequence [adapted from Large and Palmer (2002)]. **(B)** Arnold tongues diagram shows regions in parameter space in which an oscillator mode-locks to a periodic stimulus. Parameters are the ratio of signal period to oscillator period (q/p) and the coupling strength (α). Darker regions correspond to parameter values that yield faster phase-locking [adapted from Large and Kolen (1994)]. **(C)** Period adaptation of an adaptive oscillator (solid line) to a stimulus rate change (dotted line) [adapted from Large and Jones (1999)]. **(D)** An analysis showing that mode-locking is preserved in phase and period-adaptive oscillators [e.g., (Loehr et al., 2011)]. δ is an added elasticity parameter. 1:2 locking (green), 2:3 locking (orange), 1:1 locking (purple), 3:2 locking (blue) and 2:1 locking (red) are shown [adapted from Savinov et al. (2021)].

Phase oscillator models, based on circle maps, have a long history of application within dynamical systems. Given a hypothetical neural oscillation, a circle map predicts a succession of states at which events occur in a complex rhythmic stimulus. The circle map captures the fundamental hypothesis that temporal expectancies for events in a rhythmic stimulus depend on the phase of a neuronal oscillation that is driven by stimulus events, mapping time onto the phase of a neural oscillation (see Figure 4A).


(1) $$\phi_{n+1}=\phi_{n}+\frac{t_{n+1}-t_{n}}{p}-\alpha \,F\,\left(\phi_{n}\right)\,\left(m\,o\,d_{\left(-0.5,0.5\right)}\,1\right)$$

Here, *t_n_* is the time of the *n*th event, π_*n*_ is the phase of the *n*th event, and *p* is the intrinsic period of the neural oscillation. When *F*(*ϕ*_*n*_) = *sin*2πϕ_*n*_ and the stimulus is periodic, *t*_*n* + 1_ − *t*_*n*_ = *q*, this is the well-studied sine circle map (e.g., Glass and Mackey, 1988; Glass, 2001). In this case, the oscillator achieves a phase-locked state when the period of the stimulus, *q*, is not too far from the period of the oscillator, *p*. The greater the coupling strength, α, the greater the difference between the two periods can be and still achieve phase locking (a constant phase difference). Moreover, when the relative period of the oscillator and a stimulus, p/q, is near an integer ratio, the system achieves a mode-locked state. The behavior is referred to as synchronization or entrainment and is summarized in an Arnold tongues bifurcation diagram (Figure 4B; see also section “5. Neural resonance theory”).

Mode-locking is significant because musical rhythms are not just isochronous. They consist of patterned sequences of variable inter-onset intervals (IOIs; see Figure 1C), *t*_*n* + 1_ − *t*_*n*_, which contain multiple frequencies by definition. It is because of this property that Eq. 1 can model the perception of a beat at a steady tempo (frequency) in response to the multiple frequencies present in a musical rhythm.

Moreover, tempo can change in musical performances. In Eq. 1, the period *p* is fixed. However, to account for tempo changes, period adapting dynamics are introduced through a period correction term.


(2) $$p_{n+1}=p_{n}+p_{n}\,\beta \,F\,\left(\phi_{n}\right),$$

where the parameter β is the strength of the period adaptation. Together Eqs. 1, 2 constitute a period-adaptive, phase oscillator model (e.g., Large and Kolen, 1994; McAuley, 1995; Loehr et al., 2011). As illustrated in Figure 4C, period adaptation operates together with the phase entrainment to adapt to rhythms that change tempo. Period adaptation enables the system described by Eqs. 1, 2 to lock with zero phase difference to periodic rhythms different from its natural frequency (see also, Ermentrout, 1991, for a similar model of firefly synchronization). A recent analysis of the Loehr et al. (2011) model demonstrated the stability of multiple mode-locked states in the period-adapting model (Savinov et al., 2021) with and without period elasticity (Figure 4D). Extensions of this model are discussed in the sections “3.3.2. Dynamic attending and perception-action coordination” and “5. Neural resonance theory.”

#### 3.3.2. Dynamic attending and perception-action coordination

**FIGURE 5:**
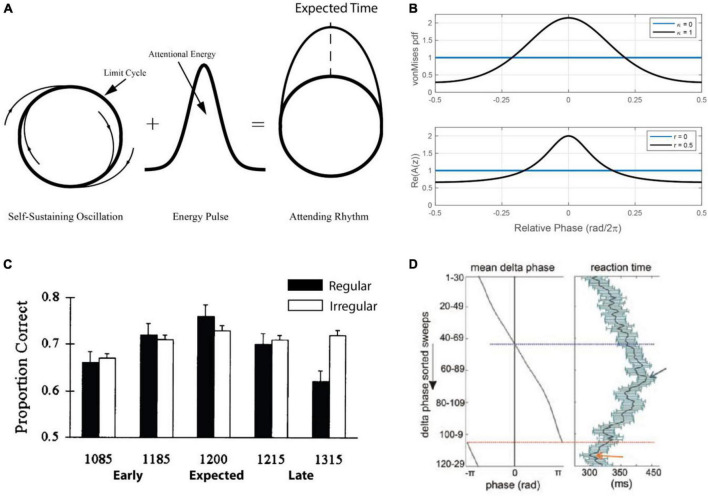
**(A)** The Dynamic Attending Theory model (DAT) consists of a limit-cycle oscillator and with a circular probability density that together model an “attending rhythm” that targets attention in time [adapted from Large and Jones (1999)]. **(B)** The energy distribution (a von Mises distribution) for the DAT model for different values of κ, the width of the distribution, which is a parameter that reflects attentional focus around the expected phase value of zero [top; adapted from Large and Jones (1999)]. The active non-linearity of the canonical model can function as the attentional pulse in a more neurally realistic model, and the amplitude (*r*) plays the same role as κ in the DAT model [bottom; see the section “5.2.2. A canonical model” and Eq. 8; (Large et al., 2010)]. **(C)** The effect of a regular versus an irregular timing cue on a pitch discrimination task. Performance is explained by a quadratic function that reflects expectation but only in the condition with a regular timing cue [adapted from Jones et al. (2002)]. **(D)** Results for a study where non-human primates carried out a visual discrimination task in a stream of visual stimuli timed around the delta-band of cortical oscillations [adapted from Lakatos et al. (2008)].

To model dynamic attending, Eqs. 1, 2 served as a quantitative model of attentional entrainment. To capture the precision of temporal expectations, an “attentional pulse” was added and defined probabilistically as


(3) $$f\,\left(\phi ,\kappa \right)=\frac{1}{I_{0}\,\left(\kappa \right)}\,e\,x\,p\,\left(\kappa \,c\,o\,s\,2\,\pi \,\phi \right)$$

which corresponds to the von Mises distribution (Figures 5A, B; see also Figure 1C). *I*_*0*_(κ) denotes the modified Bessel function of order zero. When the temporal predictions are accurate, the attentional pulse becomes narrower in time, modeling increased precision of temporal predictions. The dynamics of attentional focus is included as a third dynamical equation to estimate the concentration parameter κ of the von Mises distribution (for details, see Large and Jones, 1999; Large and Palmer, 2002). The attentional pulse enabled quantitative modeling of temporal predictions in time and pitch discrimination tasks.

This model (Figures 5A, B) inspired a number of studies showing that a predictable rhythmic context facilitates perceptual processing of a subsequent acoustic event, with highest accuracy for targets occurring in-phase with the rhythm (Jones et al., 2002; see also Large and Jones, 1999; Barnes and Jones, 2000; Jones et al., 2006). In one study (Figure 5C) pitch changes were better detected when an event occurred at an expected time, compared with early or late. This pattern of results has since been replicated in multiple behavioral studies (see Henry and Herrmann, 2014; Hickok et al., 2015; Haegens and Zion Golumbic, 2018), including in the visual domain (Correa and Nobre, 2008; Auksztulewicz et al., 2019; see also Rohenkohl et al., 2012; Rohenkohl et al., 2014). Moreover, early studies in primates (Schroeder and Lakatos, 2009; Figure 5D) and humans (Stefanics et al., 2010) directly linked facilitation of perceptual processing (e.g., reaction time) to the phase of measured neural oscillations. Such results provided critical support for underlying models of entrained neural oscillation, but emphasized the need for more physiologically realistic models of neural oscillation.

A number of studies have linked beat perception in musical rhythms and dynamic allocation of attention to motor system activity (e.g., Phillips-Silver and Trainor, 2005; Chen et al., 2008a; Fujioka et al., 2009, 2012; Grahn and Rowe, 2009; Morillon et al., 2014). One such study on perception and action showed that active engagement of the motor-system by tapping along with a reference rhythm enhances the processing of on-beat targets and suppression of off-beat distractors compared to a passive-listening condition (Morillon et al., 2014). The Active Sensing Hypothesis proposed that perception occurs actively via oscillatory motor sampling routines (Schroeder et al., 2010; Henry et al., 2014; Morillon et al., 2014). Such findings have informed the development of neural resonance theory (section “5. Neural resonance theory”; e.g., Large and Snyder, 2009; Large et al., 2015), and led to testing of the theories using perception-action coordination tasks.

Perception-action tasks provide much more fine-grained behavioral data than is possible with perception and attention tasks. Adults can synchronize in-phase at a wide range of tempos, from around 5 Hz (*p* = 200 ms; Repp, 2003; ≥8 Hz if tapping a subharmonic) down to 0.3 Hz (*p* = 3,333 ms) and probably lower (see Repp and Doggett, 2007). At slower frequencies (∼2 Hz and lower) people can either synchronize or syncopate (deGuzman and Kelso, 1991), reflecting bistable dynamics.

**FIGURE 6:**
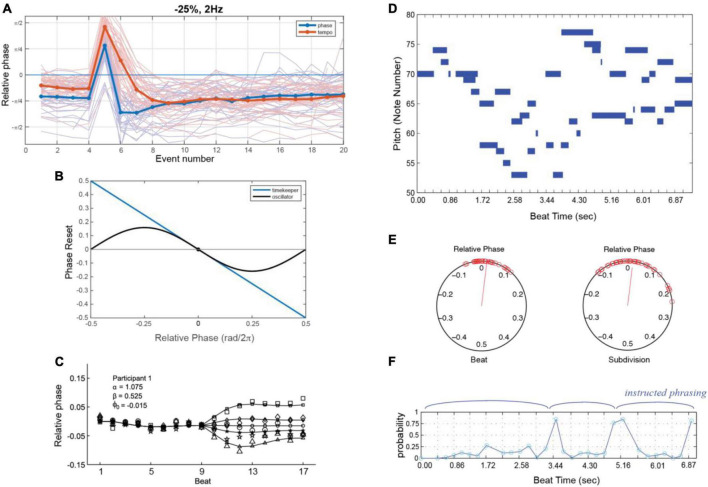
**(A)** Responses to phase (blue) or tempo (red) perturbations in a synchronization task (thin lines: individual trial; thick lines: grand average). Note that steady state synchronization also displays NMA [adapted from Wei et al. (2022)]. **(B)** The phase reset function in an oscillator model (black) as a function of relative phase with respect to the stimulus, versus the reset in a timekeeper model (blue). **(C)** An oscillator model (line with small markers) can explain asynchronies in a synchronization task where the stimulus speeds up or slows down **(B,C)** [adapted from Loehr et al. (2011)]. **(D)** Piano roll notation (as in Figure 1A) of a piano performance 3-part invention in B-flat by J.S Bach. **(E)** The relative phases of oscillators tracking beats at two different metrical levels, the main beat and a subdivision, determining attentional pulse width. **(F)** Probabilities that an event is late, computed using the attentional pulse, modeling perception of intended phrase boundaries **(E,F)** [adapted from Large and Palmer (2002)].

In one widely used paradigm, subjects synchronize finger taps (in-phase) with an isochronous rhythm. Once a steady-state phase is achieved (Repp, 2001a,b, 2003, 2008; Large et al., 2002), a phase or tempo perturbation is introduced, and relaxation back to steady-state is measured (Figure 6A). People respond quickly and automatically to phase perturbations (either permanent phase shifts or a shift of one event onset) of periodic sequences (Thaut et al., 1998; Repp, 2001b, 2002a,b, 2003; Large et al., 2002) and relaxation profiles match dynamical predictions. People also synchronize and recover from perturbations at small integer ratio frequencies (i.e., mode-locking; Large et al., 2002; Repp, 2008). People can also adapt to tempo perturbations (i.e., step changes of tempo; Large et al., 2002); however, tempo tracking is under volitional control and requires active attending (Repp, 2001a; Repp and Keller, 2004). People can also follow the periodic beat of complex musical rhythms (Large and Kolen, 1994; Large and Palmer, 2002), even those that have large changes in tempo (Rankin et al., 2009), as predicted by dynamical models (Large and Palmer, 2002; Loehr et al., 2011).

The seminal research of Haken et al. (1985) showed that anti-phase tapping with a periodic stimulus is possible due to bistable synchronization dynamics at low frequencies, but the anti-phase mode loses stability via a nonequilibrium phase transition (similar to gait transitions) if the stimulus frequency increases beyond a critical value (or bifurcation point) where monostable dynamics appear. Syncopation becomes difficult as the stimulus tempo increases even for trained musicians (e.g., Keller and Repp, 2005; Repp and Doggett, 2007). deGuzman and Kelso (1991) showed a phase model, similar to Eq. 1 but with a slightly different coupling function, could capture their findings.

The models considered here also allow for exploration of conceptual questions such as the relationship between dynamical systems approaches and information processing approaches. A dynamical systems approach is meant to embody the notion that a neural oscillator adapts its period to an external (forcing) stimulus, as in Eqs. 1, 2. Information processing models suggest that internal time-keepers measure and linearly track time intervals (e.g., Wing and Kristofferson, 1973; Mates, 1994a,b; Vorberg and Wing, 1996; van der Steen and Keller, 2013). One analysis showed that the phase oscillator model of Eqs. 1, 2 is formally similar to a linear time-keeper model (Jagacinski et al., 2000; Loehr et al., 2011), such that for periodic (isochronous) stimuli and small temporal perturbations, the predictions are nearly identical (Figure 6B). However, when responding to larger perturbations, linear models incorrectly predict symmetry in speeding up versus slowing down, whereas oscillator models correctly predict an asymmetry (Figure 6C; Loehr et al., 2011; see also Bose et al., 2019).

The critical difference, however, is that linear time-keeper models capture only 1:1 synchronization, whereas oscillator models capture mode-locking as well (Loehr et al., 2011). Mode-locking implies the ability to coordinate with complex music-like rhythms (Figures 6D–F), thus modeling the perception of beat, meter, and phrasing. In one study, oscillators entrained to complex rhythms in classical piano performances (e.g., 3-part invention by JS Bach; shown in Figure 6E) at multiple metric levels (Figure 6E). The precise temporal expectancies embodied in the attentional pulse were then used to model the perception of phrase boundaries (Figure 6F), which are marked by slowing of tempo (i.e., phrase-final lengthening; see Large and Palmer, 2002).

## 4. Inference models of rhythm perception

A different computational approach to beat perception draws on the theory of the *Bayesian Brain* and treats rhythm perception as a process of probabilistic inference. Rather than asking what neural mechanisms give rise to the percept of beats, it asks how we can understand human rhythm perception as part of the brain’s general strategy for making sense of the world. While this question is essentially a cognitive one, models that address this question may begin to guide and constrain models of the neural mechanisms that must ultimately undergird them.

### 4.1. The Bayesian Brain and predictive processing

One point of entry into the Bayesian Brain theory is the proposition that organisms are well served by producing internal dynamics corresponding to (or “representing”) the dynamic states and unknown parameters of survival-relevant processes in the world around them, which they can then use to predict what will happen next. A formal version of this claim is called the Free Energy Principle (Friston, 2010). In the case of rhythm perception, this representation may encompass the static parameters and dynamic states of any process that determines the timing and sequence of auditory events: for example, the nature of an underlying repetitive metrical pattern, the momentary phase and tempo of that pattern, and the number and identities of distinct agents generating the pattern. These representations can then inform predictions of upcoming auditory events and guide the entrainment of movements. Representing these variables might be survival-relevant by, for example, allowing groups of humans to coordinate their steps and actions rhythmically.

An attempt to represent the dynamic variables and parameters underlying the generation of a rhythm encounters multiple levels of ambiguity. A given rhythmic surface may admit multiple possible organizing metrical structures (see, e.g., Figure 1A), may be generated by one or by multiple agents, and may be corrupted by temporal irregularity or noisy sensory delays that obscure the underlying temporal structure. An idealized approach to coping with this ambiguity is given by Bayes Rule, a theorem from probability theory that describes an optimal method of incorporating noisy, ambiguous sensory observations into probabilistic estimates of underlying hidden (not directly observable) variables. Bayes Rule transforms a “prior” (pre-observation) distribution *P*(*S*) over a hidden state *S* into a “posterior” (post-observation) distribution *P*(*S*|*O*) that has incorporated an observation *O*. It does so using a “likelihood” function *P*(*O*|*S*) that describes the probability of observation *O* given any of the possible values of the hidden state *S*. The likelihood function can be understood as a model of how the hidden state generates observations, or a *generative model*.

The theory of the Bayesian Brain proposes that the brain mimics the application of Bayes Rule as it integrates sensory data into representations of the world, where generative models are implicitly learned through a lifetime (or evolutionary history) of interaction with the world. Note that the application of Bayes Rule does not entail a Bayesian interpretation of probability more generally – the essential contribution of Bayes rule in this context is a formal method of weighting the influence of each new observation by its precision relative to the precision of estimates preceding the observation.

The theory of Predictive Processing elaborates on the Bayesian Brain theory. It posits that the brain may be approximating Bayes Rule by continuously changing representations to minimize the difference between actual sensory input and the sensory input predicted based on those representations (the “prediction error”; Friston, 2005). Learning generative models can proceed similarly, by gradually changing them to minimize prediction error on time scales ranging from minutes to years, though some aspects of the generative models may be innate. Importantly, unlike in static Bayesian models of perception where statistically learned priors are combined with single observations to yield percepts, Predictive Processing proposes that inference proceeds dynamically, with the posterior after one observation acting as the prior for the next.

### 4.2. Modeling rhythm perception as inference

The qualitative groundwork for an inference theory of rhythm was laid by Lerdahl and Jackendoff (1996), who framed music cognition in terms of inference. In their view, the listener brings a set of musical intuitions, some of them learned, to each musical experience, and uses them to infer the latent structure underlying the musical surface (including the rhythmic surface). In their model, they treated meter as a hidden state to be inferred and upon which to base auditory timing predictions. Vuust, Witek, and later their coauthors (Vuust and Witek, 2014; Vuust et al., 2018) applied ideas from Predictive Processing to the perception of musical meter, positing that the perception of meter is determined through variational Bayesian inference, i.e., prediction error minimization, and arguing that this perspective accounts for various experimental results.

Perhaps the first fully mathematically specified inference model of rhythmic understanding took as the inferred hidden state the number of distinct processes generating a combination of jittered metronomic stimuli and used this model to account for participant tapping behavior, which synchronized differently depending on the phase proximity and jitter of the superimposed streams (Elliott et al., 2014). More recently, a model was proposed for two-participant synchronized tapping in which each tapper inferred whether the self-generated and other-generated taps could best be explained with two separate predictive models or one (“self-other integration”; Heggli et al., 2021).

### 4.3. PIPPET: a model of stimulus phase inference

The PIPPET (Phase Inference from Point Process Event Timing) model (Cannon, 2021) attempts to characterize rhythm perception as a formal probabilistic inference process in continuous time. In its simplest incarnation, it describes an observer’s process of inferring the phase of a cyclically patterned stimulus given a known tempo and meter.

#### 4.3.1. Specifications

PIPPET starts with a generative model of rhythms based on a specific underlying metrical pattern, e.g., the cycle of the beat or of a group of multiple beats. Note that this generative model is not necessarily the way in which an actual stimulus is generated; instead, it describes the beliefs or expectations of the observer for the purposes of creating a representation of the rhythm and predicting future sound events. According to this generative model, the unfolding of a rhythmic stimulus is driven by a dynamic “phase” variable *ϕ*_*t*_ representing progress through the metrical pattern at time *t*, e.g., the momentary phase (on the circle) of an ongoing beat at time *t*. *ϕ*_*t*_ is expected to progress at a steady rate θ over time (corresponding to the known tempo), but with Brownian noise σ*W*_*t*_ representing the fact that even a perfectly steady rhythm will seem slightly irregular to an observer with noisy internal timekeeping:


$$d\,\phi =\theta \,d\,t+\sigma \,d\,W_{t}$$

**FIGURE 7:**
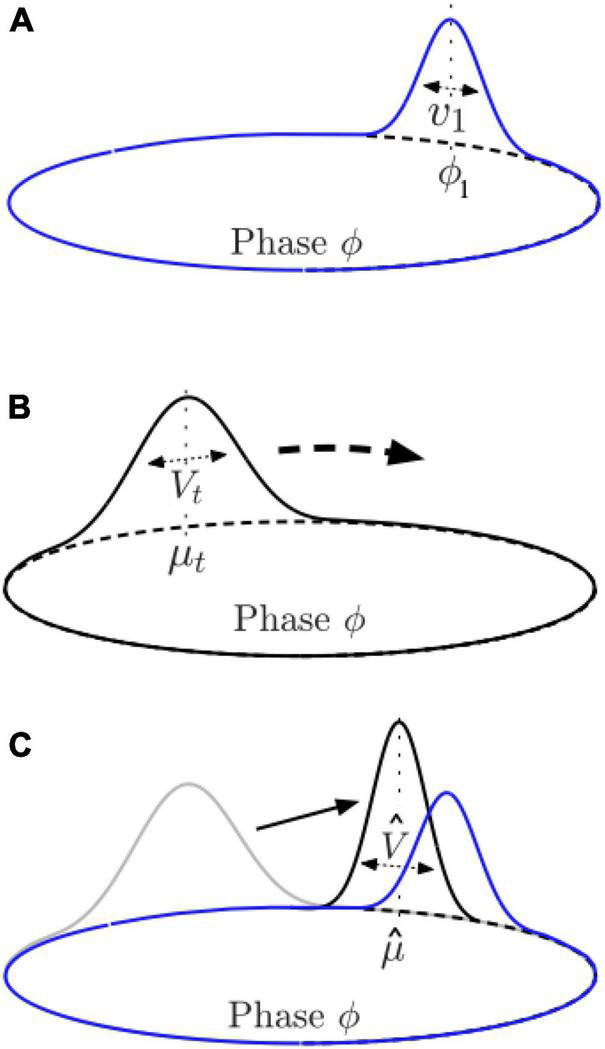
Illustration of components of the PIPPET model. **(A)** In PIPPET, rhythmic expectations are represented with one or more Gaussian peaks on the circle, with means representing phases where events are expected, e.g., *ϕ*_1_, and variances representing the temporal precision of those expectations, e.g., *v_1_*. **(B)** The observer approximates the phase of the cyclically patterned stimulus with a Gaussian distribution over the circle, with mean (estimated phase) μ_*t*_ and variance (uncertainty) *V_t_* at time *t*. In the absence of sound events or strong expectations, μ_*t*_ moves steadily around the circle while *V_t_* grows. **(C)** At a sound event, μ_*t*_ resets to $\hat{\mu }$, moving the estimated phase closer to a nearby phase at which events are expected, and *V_t_* resets to $\hat{V}$, adjusting the certainty about phase as appropriate.

The observer is assumed to know or have already identified an underlying metrical structure or “*expectation template*” for the rhythm (e.g., duple or triple subdivisions of a steady beat). This may be drawn from a library of learned expectation templates; we discuss the process of choosing a template below. The metrical pattern expected by the observer is represented by a function λ(*ϕ*) from stimulus phase to the observer’s belief about the probability of a sound event at that phase. This function consists of a sum of a constant λ_0_, representing the probability of non-metrical sound events, and a set of Gaussians, each with a mean *ϕ*_*j*_ representing a characteristic phase at which the observer expects events to be most probable, a scale λ_*j*_ representing the probability of events associated with that characteristic phase, and a variance *v_j_* related to the temporal precision with which events are expected to occur at that phase (Figure 7A):


$$\lambda \,\left(\phi \right)=\lambda_{0}+\sum_{j}\frac{\lambda_{j}}{\sqrt{2\,\pi \,v_{j}}}\,e^{-\frac{\left(\phi -\phi_{j}\right)}{2\,v_{j}}}$$

According to the generative model, rhythmic events are produced as an inhomogeneous point process with rate λ(*ϕ*_*t*_). As a map over cycle phase representing the position and precision of temporal expectancy, this function serves a very similar purpose to the “attentional pulse” in DAT models described above.

The observer uses the generative model to continuously infer the stimulus phase *ϕ*_*t*_, approximating a full distribution over possible phases at time *t* with a Gaussian distribution with mean μ_*t*_ and the variance *V_t_* (Figure 7B):


$$P\left(\phi_{t}=\phi \right)=\frac{1}{\sqrt{2\,\pi \,V_{t}}}e^{-\frac{{\left(\phi -\mu_{t}\right)}^{2}}{2\,V_{t}}}$$

The parameters μ_*t*_ and *V_t_* of this distribution are adjusted to make the best possible estimate of phase over time, handling various sources of timing and event noise through a variant on continuous-time Kalman filtering (Bucy and Joseph, 2005) that amounts to a continuous application of Bayes rule, and that can be described by the equations below.

At any time *t*, let Λ denote the observer’s expectancy (or “subjective hazard function”) for sound events, defined as


$$\Lambda =\sum_{j}\Lambda_{j}\,\text{where}\,\Lambda_{j}=\lambda_{j}\,e^{-\frac{{\left(\phi_{j}-\mu_{t}\right)}^{2}}{V_{t}+v_{j}}}.$$

Define auxiliary variables $\hat{\mu }$ and $\hat{V}$:


μ^=∑jΛjΛ⁢μj^⁢where⁢μi^=Vt-1⁢μt+vj-1⁢ϕjVt-1+vj-1

V^=∑jΛjΛ⁢(Vj^+(μ^-μj^)2)⁢where⁢Vj^=1Vt-1+vj-1.

A rule for the continuous evolution of the variables μ_*t*_ and *V_t_* between events can be defined:


$$\frac{d\,\mu }{d\,t}=\theta +\Lambda \,\left(\hat{\mu }-\mu_{t}\right)$$

$$\frac{d\,V}{d\,t}=\sigma^{2}+\Lambda \,\left(\hat{V}-V_{t}\right)$$

as well as a rule for their instantaneous reset at any event time *t* (Figure 7C):


$$\mu_{t}\to \hat{\mu }\,\text{and}\,V_{t}\to \hat{V}.$$

The rapid resetting of μ_*t*_ can be understood as a partial correction of inferred phase in response to event timing prediction error. Although the theoretical roots of PIPPET are very different from those of the oscillator models described here, the resulting dynamics are rather similar: the dynamics of μ_*t*_ are closely analogous the phase dynamics of a pulse-forced DAT model, and the dynamics of *V_t_* behave somewhat like the radial dynamics in a damped oscillator like those that appear in neural resonance models (with larger *V_t_* corresponding to a smaller radius), as described in the next section.

#### 4.3.2. Behavior, implications, and extensions

**FIGURE 8:**
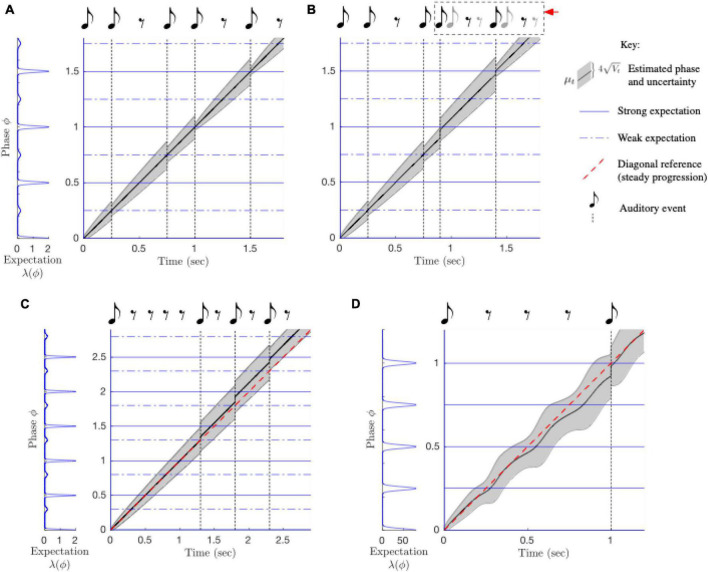
Behavior of the PIPPET model. **(A)** The PIPPET algorithm continuously estimates the phase (drawn here on the real line rather than the circle) underlying a syncopated rhythm. It does so by continuously evaluating the rhythm in light of an expectation template of times at which events are more or less strongly expected (blue). Here, the template is chosen *a priori*; in an extended model, it may be inferred from among a library of such expectation templates. For this relatively simple rhythm, estimated phase advances steadily. Uncertainty grows through rests since they provide no information about the exact phase of the stimulus cycle. Uncertainty decreases each time a sound aligns with a moment of sound expectation, and decreases substantially when a sound aligns with the strong and precise expectation on the beat, e.g., at 1 s. **(B)** Phase tracking is robust to timing perturbations in the rhythm. Immediately following a phase shift (at 1 s), phase uncertainty *V_t_* increases, but then it is reduced when the adjusted phase is used to accurately predict a strongly expected event (at 1.4 s). **(C)** The listener may expect a steady stream of events or may learn irregular patterns of expectations. Here, the listener expects uneven (swung) eighth notes. For this well-timed but excessively syncopated example rhythm, excessive syncopation leads to a failure of phase tracking (straying from the diagonal) similar to that observed for high syncopation in humans. **(D)** Strong expectations that are not met with auditory events (at 0.25, 0.5, and 0.75 s) cause the advance of estimated phase to become irregular. As a result, an auditory event at 1 s seems early.

PIPPET tracks stimulus phase and anticipates sound events through steady rhythms (Figure 8A) and rhythms that are jittered or perturbed in time (Figure 8B). The model accounts for listeners’ tendency to perceptually shift the phase of a beat if the rhythm is overly syncopated: when phase uncertainty *V_t_* is large, $\hat{\mu }$ is strongly influenced by strong phase-specific expectations even if the sound event occurs well before or after that expectation (Figure 8C). Unlike other models of rhythm perception, PIPPET accounts for the empirically observed tendency to perceive rhythms with unexpected omissions as speeding up (Repp and Bruttomesso, 2009): the $\Lambda \,\left(\hat{\mu }-\mu_{t}\right)$ term in the continuous evolution of μ_*t*_ produces a slowing of estimated phase as it approaches a phase where events are strongly expected, resulting in the next sound seeming early (Figure 8D).

A key aspect of the model that follows from the formulation of the problem as probabilistic inference is *V_t_*, the continuous estimate of the participant’s certainty about the rhythm’s phase. This work predicts that a similar estimate should be physiologically represented in the brain alongside an estimate of the phase of the beat cycle. The model is agnostic to the neural nature of μ_*t*_ and *V_t_*, and could therefore be compatible with a range of physiological mechanisms.

One exciting aspect of this model, and of probabilistic inference approaches more generally, is that they can be used to specify an exact degree of mismatch between expectations and reality. A convenient measure of this mismatch is the information-theoretic surprisal: if the probability of an occurrence is *p*, the surprisal associated with it is −*log*(*p*). Thus, the surprisal associated with a sound event is −*log*(Λ*dt*), a function of the subjective hazard rate, i.e., the overall degree of expectancy. This is closely related to the prediction error signal thought to drive Bayesian inference in the theory of Predictive Processing.

**FIGURE 9:**
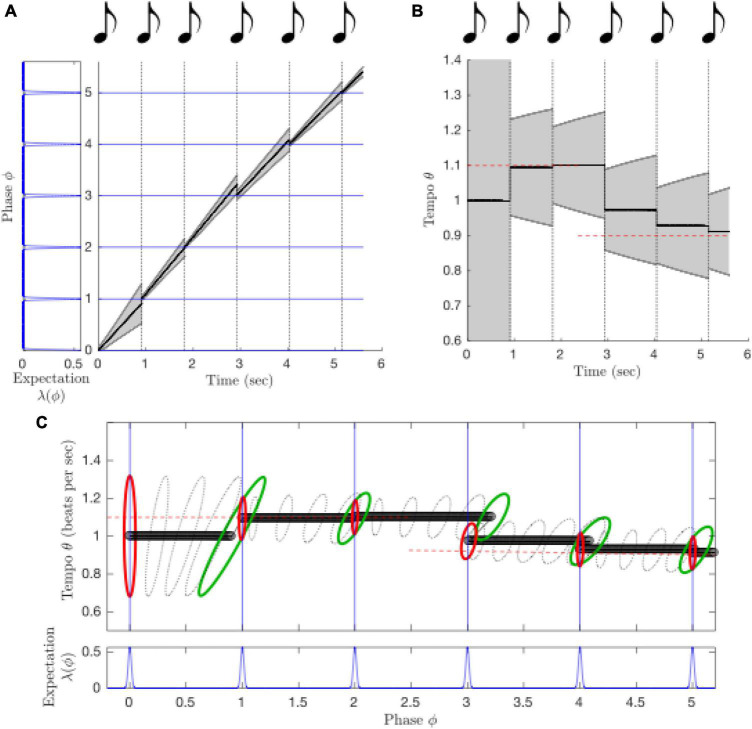
The extended PIPPET model infers both phase and tempo over the course of a tempo change from 1.1 beats per second to 0.9 beats per second. **(A)** When the sequence of sound events slows down, the advance of estimated phase slows as well. See previous figure key. **(B)** This is because tempo (and tempo uncertainty) are also being inferred as the sequence unfolds, and the estimated tempo drops when the rate of events slows. Initial and final tempo are marked in dashed red lines. **(C)** Phase and tempo can be inferred simultaneously because the model tracks a joint distribution over phase (*X*-axis, projected from the circle onto the real line) and tempo (*Y*-axis). The contours represent level sets of a multivariate Gaussian distribution over phase/tempo state space, strobed over time. At each event, the prior distribution (red) updates to a posterior (green) that incorporates a likelihood function based on the phases at which events are expected.

PIPPET can be extended to include inference of stimulus tempo as well as phase: when a dynamic tempo variable is incorporated into the generative model of rhythm, a dynamic probabilistic inference process can continuously estimate a joint distribution over phase and tempo (Figure 9). Further, it has recently been extended to include simultaneous inference about which of a library of templates is relevant to the current rhythm (Kaplan et al., 2022). In this extension, the collection of metrical patterns that the listener has been enculturated to expect determines how they interpret, tap along with, and reproduce the rhythmic surface, as has been demonstrated in experiment (Jacoby and McDermott, 2017).

The PIPPET model takes an interesting stance on the continuation of a beat. Continued rhythmic input is necessary in order for the estimated distribution over stimulus phase not to decay to a uniform distribution. However, that input can be self-generated. If self-movement, e.g., stepping or finger-tapping, is actuated based on the inferred rhythmic structure, then the feedback from this movement can take the place of the stimulus and keep the phase inference process going in a closed loop of action and rhythm perception. Alternatively, auditory imagery, presumably generated elsewhere in the brain and treated as input to PIPPET, could take the place of motor feedback.

There has not yet been a published attempt to carefully fit PIPPET’s parameters to rhythm perception data. However, such an attempt is not unrealistic. The expectation template could be estimated by manipulating timing at various levels of metrical hierarchy and observing the effect on the timing of a subsequent tap indicating the next expected beat (e.g., Repp and Doggett, 2007), and the rate of accumulation of temporal uncertainty between events could be worked out by doing a similar experiment across multiple inter-beat intervals.

The Bayesian Brain perspective on rhythm perception will not, in itself, reveal neural mechanisms of beat tracking and anticipation. However, it has already demonstrated its potential to highlight nuances of rhythm perception and model them at a high level, providing guidance to the search for neural substrates. Further, by identifying the cognitive significance of dynamic variables as “representations” of hidden underlying processes, this dynamic inference approach may provide a bridge from basic mechanisms to complex musical behaviors that are easiest to specify in terms of recognition of and interaction with musical structure.

## 5. Neural resonance theory

While Bayesian approaches model rhythm at the behavioral level, in this section we explore the hypothesis that physiological oscillations in large scale brain networks underlie rhythm perception. We then ask what additional behavioral and neural phenomena may be explained by this hypothesis. But this approach brings with it significant challenges. Although the mechanisms of spiking and oscillation in individual neurons have been well known for some time (Hodgkin and Huxley, 1952; Izhikevich, 2007), there remain a variety of approaches for understanding emergent oscillations within large groups of neurons (see e.g., Ermentrout, 1998; Buzsáki, 2004; Wang, 2010; Breakspear, 2017; Senzai et al., 2019).

While one type of research goal involves understanding how oscillations at different levels of organization are generated, another important goal is to understand how emergent oscillations in large scale brain networks relate to perception, action, and cognition at the behavioral level (Kelso, 2000). The Neural Resonance Theory (NRT) approach begins with the observation that models of neural oscillation share certain behavioral characteristics that can be captured in generic models called *normal forms*, or *canonical models*. It hypothesizes that the properties found at this level of analysis are the key to explaining rhythmic behavior.

Neural Resonance Theory refers to the hypothesis that generic properties of self-organized neuronal oscillations directly predict behavioral level observations including rhythm perception, temporal attention, and coordinated action. The theory’s predictions arise from dynamical analysis of physiological models of oscillations in large-scale cortical networks (see Hoppensteadt and Izhikevich, 1996a,b; Breakspear, 2017). As such, NRT models make additional predictions about the underlying physiology, including local field potentials, and parameter regimes of the underlying physiological dynamics.

### 5.1. Basic elements of NRT

#### 5.1.1. Neural resonance

Nonlinear *resonance* refers broadly to synchronization, or entrainment, of (nonlinear) neural oscillations (Pikovsky et al., 2001; Large, 2008; Kim and Large, 2015). Although terms like entrainment are sometimes used in the empirical literature to refer merely to phase-alignment of neural signals, these terms are used here in a stricter physical (or dynamical) sense, enabling predictions about behavior and physiology that arise from dynamical analysis of nonlinear oscillation.

#### 5.1.2. Mode locking

Importantly, neural resonance predicts more than phase-alignment with rhythmic stimuli; it predicts *mode-locking* of neural oscillations. Mode-locking, in turn, predicts structural constraints in perception, attention, and coordinated action. Mode-locking can explain, for example, how we perceive a periodic beat in a complex (multi-frequency) rhythm (Large et al., 2015), whereas phase-alignment in linear systems cannot (see e.g., Pikovsky et al., 2001; Loehr et al., 2011).

#### 5.1.3. Neural plasticity

Synaptic plasticity is an important means by which the brain adapts and learns through exposure to environmental information. Here we describe two primary mechanisms by which adaptation may occur: *Hebbian learning* via synaptic plasticity, and behavioral timescale adaptation of individual oscillator parameters, such as natural frequency.

#### 5.1.4. Transmission delay

*Transmission delays* are ubiquitous in the brain, representing another generic feature of neural dynamics. But how can we perform synchronized activities, such as play music together, or anticipate events in a rhythm in the presence of time delays? Recent work suggests that such behavioral and perceptual feats take place not in spite of time delays, but because of them (Stephen et al., 2008; Stepp and Turvey, 2010). While seemingly counter-intuitive, dynamical systems can anticipate or expect events in the external world to which it is coupled, without a need to explicitly model the environment or predict the future.

### 5.2. NRT: oscillatory models from series expansions

Phase models based on circle map dynamics, introduced in section “3. Models for time-keeping, beat generation and beat perception,” have been used to describe phenomena including beat perception (Large and Kolen, 1994), dynamic attending (Large and Jones, 1999), perception-action coordination (deGuzman and Kelso, 1991), and tempo invariance (Large and Palmer, 2002). Inclusion of period correcting dynamics (e.g., Large and Jones, 1999; Large and Palmer, 2002) allowed these models to be robust to stimuli that change tempo. In some models, an “attentional pulse” described when in time events in an acoustic stimulus were expected to occur as well as the precision of temporal expectations. A related “canonical” model was derived from a physiological model of oscillation in cortical networks (Wilson and Cowan, 1973; Hoppensteadt and Izhikevich, 1996a,b; Large et al., 2010), and includes both amplitude and phase dynamics. The inclusion of realistic amplitude dynamics enriches the behavioral predictions of phase models and has also led to models that include Hebbian learning and neural time-delays.

#### 5.2.1. Relation to mechanistic cortical oscillation models

**FIGURE 10:**
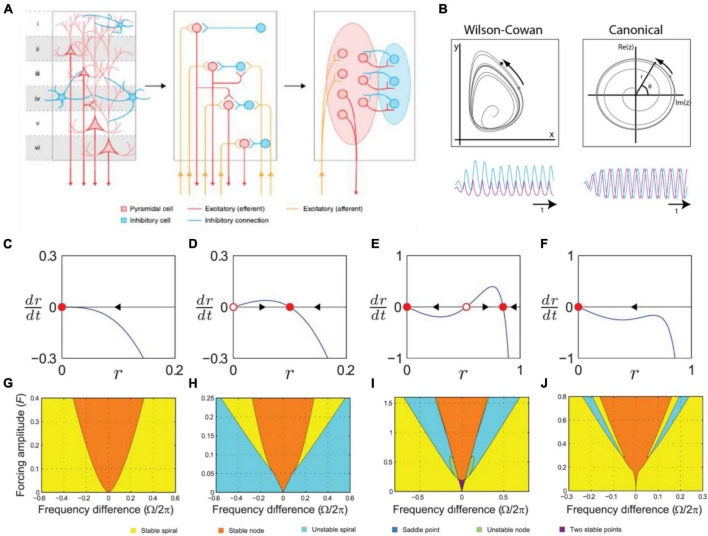
**(A)** The left panel shows pyramidal and inhibitory neurons present and interacting across a cortical column. The center panel shows how a traditional spiking neural model treats each neuron as a unit that is individually modeled. The right panel shows a neural mass model (NMM), where population dynamics are averaged to a low-dimensional differential equation for each class of neurons [modified from Breakspear (2017)]. **(B)** The oscillatory dynamics of two-dimensional Wilson-Cowan (left) and a canonical oscillator (right) model, driven by a periodic sinusoidal stimulus [adapted from Large (2008)]. **(C–F)** Amplitude vector field for a canonical oscillator in a critical Hopf regime, **(D)** a supercritical Hopf regime, and **(E,F)** saddle-node bifurcation of periodic orbits regimes. Attractors and repellers are indicated by red dots and red circles, respectively. Arrows show direction of trajectories [adapted from Kim and Large (2015)]. **(G–J)** Stability regions for a canonical oscillator as a function of sinusoidal forcing strength in panel **(G)** a critical Hopf regime, **(H)** a supercritical Hopf regime, **(I)** saddle-node bifurcation of periodic orbits regimes. Colors indicate stability type [adapted from Kim and Large (2015)].

Phase models represented important steps forward in thinking about what kind of neural dynamics may underlie dynamic attending and perception-action coordination. However, the formalism described in section “3.3.1. Circle-map phase oscillator models,” specifically the absence of amplitude dynamics, limits applicability to neural and behavioral data. Alternatively, neural mass models (also called excitation-inhibition, or E-I models, e.g., Wilson and Cowan, 1973; Hoppensteadt and Izhikevich, 1996a) characterize neural oscillations in networks of interacting excitatory and inhibitory neural populations. Models of large-scale brain dynamics (larger than a single neural oscillation) can then be based on the study of self-organized oscillation in collective neural behavior (see Breakspear, 2017 for an overview) in which oscillations have both amplitude and phase (e.g., Large et al., 2010; see Figures 10A, B). Below, we describe a derivation of a canonical model from a neural mass model, and then expand this into a network model with learning and address the role of time delays.

Neural mass models approximate groups of neurons by their average properties and interactions. One example is the well-known Wilson-Cowan model (Wilson and Cowan, 1973), which describes the dynamics of interactions between populations of simple excitatory and inhibitory model neurons. Both limit cycle behavior, i.e. neural oscillations, and stimulus-dependent evoked responses are captured in this model.


(4) $$\frac{d\,u}{d\,\tau }=-u+S\,\left(\rho_{u}+a\,u-b\,v+x_{u}\,\left(\tau \right)\right)$$

$$\frac{d\,v}{d\,\tau }=-v+S\,\left(\rho_{v}+c\,u-d\,v+x_{v}\,\left(\tau \right)\right)$$

Here, *u* is the firing rate of an excitatory population, and *v* is the firing rate of an inhibitory population. *a, b, c*, and *d* are intrinsic parameters, ρ_*u*_ and ρ_*v*_ are bifurcation parameters, and *x*_*u*_(τ) and *x*_*v*_(τ) are afferent input to the oscillator.

#### 5.2.2. A canonical model

On the surface, neural mass models appear quite distinct from the sine circle map used in section “3.3.1. Circle-map phase oscillator models.” However, there is a systematic relationship. One approach to understanding their behavior is to derive a normal form. When the neural mass model is near a Hopf bifurcation, we can reduce it to the Hopf normal form via a coordinate transformation and a Taylor expansion about a fixed point (equilibrium), followed by truncation of higher order terms after averaging over slow time (Ermentrout and Cowan, 1979; Hoppensteadt and Izhikevich, 1997).


$$\frac{d\,z}{d\,t}=z\,\left(a+b_{1}\,{\left|z\right|}^{2}\right)+x\,\left(t\right)+H.O.T.$$

where *z* = *re^iϕ^* is complex-valued oscillator state, *a* = α + *i*ω and *b*_1_ = β_1_ + *i*δ_1_ are coefficients to the linear and cubic terms, respectively, *x(t)* is an external rhythmic input that may contain multiple frequencies, and *t* = ετ is slow time, and H.O.T. represents higher order terms. Eq. 5 can then be transformed to separately describe the dynamics of amplitude *r* and phase ϕ:


(5) $$\frac{d\,r}{d\,t}=r\,\left(\alpha +\beta_{1}\,r^{2}\right)+x\,\left(t\right)\,c\,o\,s\,\phi$$

(6) $$\frac{d\,\phi }{d\,t}=\omega +\delta \,r^{2}-\frac{x\,\left(t\right)}{r}\,s\,i\,n\,\phi$$

In this formulation, the parameters are behaviorally meaningful, α is the bifurcation parameter, ω is the natural frequency, β_1_is nonlinear damping, and δ is a detuning parameter that captures the dependence of frequency on amplitude. This model is systematically related to the phase model, assuming a limit cycle oscillation (α ≫ 0) and discrete input impulses (see Large, 2008).

Normal form models are useful in their own right, for example as models of outer hair cells in the cochlea (e.g., Eguíluz et al., 2000). However, by truncating higher order terms important properties of the neural models are lost, most importantly mode-locking, which is critical in the NRT framework. However, using the same principles as normal form analysis, a fully expanded canonical model for a neural mass oscillation near a Hopf bifurcation can be derived (Large et al., 2010). For example, the following expanded form contains the input terms for all two-frequency (k:m) relations between an oscillator and a sinusoidal input:


$$\frac{d\,z}{d\,t}=z\,\left(a+b_{1}\,{\left|z\right|}^{2}+b_{2}\,{\left|z\right|}^{4}+\ldots \right)+c_{11}\,x+c_{12}\,x\,\bar{z}+c_{13}\,x\,{\bar{z}}^{2}+\ldots$$

(7) $$c_{21}\,x^{2}+c_{22}\,x^{2}\,\bar{z}+c_{23}\,x^{2}\,{\bar{z}}^{2}+\ldots$$

In the original expanded form in Large et al. (2010), the relative strengths of high-order terms are expressed by powers of the parameter ∈. Eq. 8 is a rescaled form without ∈ in which oscillation amplitude is normalized (|*z*| < 1; Kim and Large, 2021). This model displays rich dynamics and has several interesting properties that make it useful for modeling and predicting rhythmic behaviors. The fully expanded model can be decomposed to study specific properties, and to create minimal models of empirical phenomena.

In terms of the input expansion, each term in Eq. 8, which takes the form $x^{k}\,{\bar{z}}^{m-1}$, corresponds to a different *k*:*m* mode-lock: 1:1, 1:2, 1:3, …, 2:1, 2:2, 2:3, … This leads to analyses like that in Kim and Large (2019). Eq. 8 includes an infinite series of input terms, each for a different frequency relation, because it is assumed that the stimulus frequency is unknown. When the frequency relation is known, the canonical model can include only one input term (called resonant monomial). Figure 10B compares an oscillation in the Wilson Cowan model with an oscillation in the fully expanded model. Note that the limit cycle is transformed into a circle, making behavior-level analyses straightforward (Kim and Large, 2015). Four different parameter regimes are available for describing different types of driven behavior (Figures 10C-F). Note that the canonical model can display a Hopf bifurcation and also a saddle-node bifurcation of periodic orbits (also called a double limit cycle bifurcation), as E-I models do (Hoppensteadt and Izhikevich, 1997; Kim and Large, 2015). Moreover, the amplitude dynamics changes the phase dynamics (Figures 10G-J; see Kim and Large, 2015).

The full expansion of the E-I model has two infinite series, one for the intrinsic dynamics and another for the input, assuming that *b*2 = *b*3 = … = *b*_*n*_, and c = *c*11 = *c*12 = … = *c*21 = … = *c*_*km*_. Both can be summed and written in the form:


(8) $$\frac{d\,z}{d\,t}=z\,\left(a+b_{1}\,{\left|z\right|}^{2}+b_{2}\,\frac{{\left|z\right|}^{4}}{1-{\left|z\right|}^{2}}\right)+c\,\frac{x}{1-x}\,\frac{1}{1-\bar{z}}$$

which makes it possible to simulate the full canonical model directly. This is not the most general form, but suffices to fit a wide range of data. These properties combine to provide a model that can be analyzed to make predictions about rhythmic behavior, as described below.

In terms of modeling dynamic attending, the canonical model provides an important link to the neural level. The phase model (Eq. 1) is systematically related to the phase of the canonical model (Large, 2008) but the canonical model includes an amplitude dimension. Amplitude dynamics provides multiple parameter regimes for entrained oscillation beyond limit cycle dynamics, which have often been assumed to be the only possibility in empirical research (e.g., Lakatos et al., 2019). Moreover, the continuous time model includes an “active nonlinearity,” $A\,\left(z\right)=R\,e\,\left(1/\left(1-\bar{z}\right)\right)$ (cf., Eq. 9; see Large et al., 2010) which depends on the amplitude of the oscillation. As the amplitude of the oscillation increases, reflecting successful predictions, *A*(*z*) becomes peakier (see Figure 5B, bottom), similar to the attentional pulse. Moreover, *A*(*z*) can actually be considered a circular probability density function – akin to the von Mises distribution – because the area under the curve for one cycle is unity. In other words, an attentional pulse function arises in the derivation of the canonical oscillator from neural mass models. Additionally, because it appears in the input term, it also functions as a temporal receptive field (Large and Kolen, 1994; Large and Palmer, 2002).

Behavioral timescale adaptation of frequency has been studied in various neural oscillator models (Righetti et al., 2006; Roman et al., 2019). Frequency adaptation is similar to period adaptation in DAT models, but is more appropriate to the differential equation framework. Lambert et al. (2016; Eq. 10) proposed extending neural resonance models to include a slow elastic return to the preferred natural frequency, which helps with stability, giving the following form:


(9) $$\frac{d\,\omega }{d\,t}=-c_{\omega }\,x\,\left(t\right)\,A\,\left(z\right)/r-c_{e}\,\frac{\omega -\omega_{o}}{\omega }$$

where *c*_ω_ is adaptation strength, *c*_*e*_ is elasticity strength, ω_0_ is preferred natural frequency, and *x(t)* is an external rhythmic input that may contain multiple frequencies. Thus, the single canonical oscillator with frequency adaptation can track rhythms that include phase and frequency perturbations as with earlier models (i.e., Large and Jones, 1999).

#### 5.2.3. Networks and learning

**FIGURE 11:**
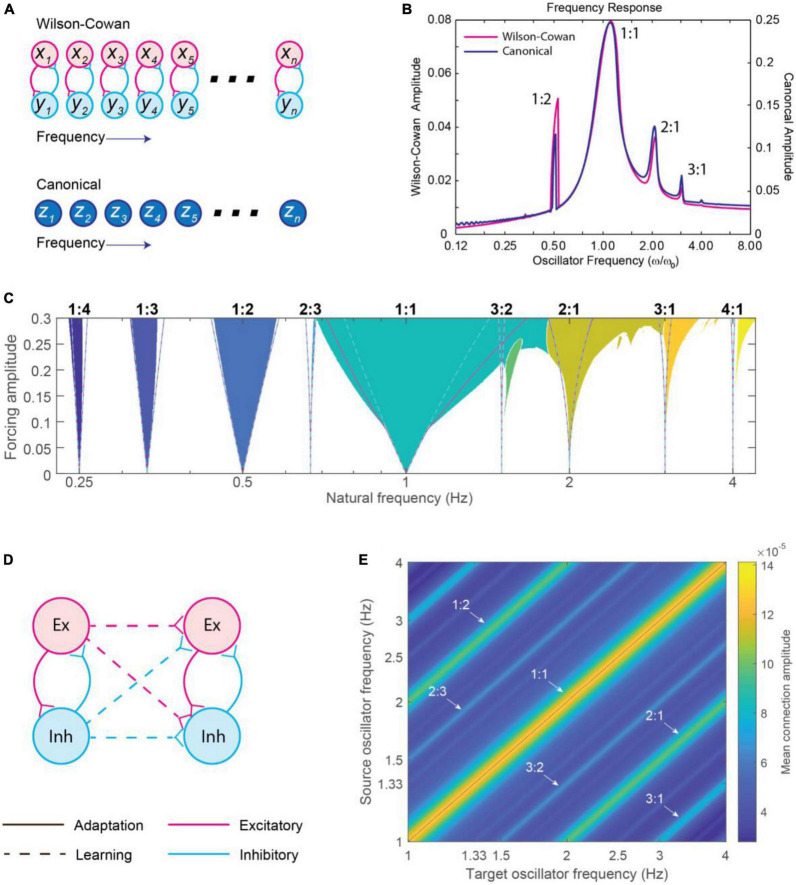
Network models, mode-locking, and learning. **(A)** Two gradient frequency neural networks: a Wilson-Cowan network (an E-I type neural mass model, top) and a canonical network (bottom). **(B)** Despite different levels of mathematical abstraction, both network models show qualitatively identical dynamics in response to a sinusoidal input, with strong resonances at the stimulus frequency (1:1) and its harmonics (2:1 and 3:1) and a subharmonic (1:2). From Large et al. (2010). **(C)** The Arnold tongues (resonance regions) for nonlinear resonances in a canonical network driven by a sinusoidal input. From Kim and Large (2019). **(D)** Synaptic connections within (vertical) and between (horizontal and diagonal) oscillatory units in a coupled neural mass model. **(E)** Connections formed between canonical oscillators in a gradient frequency network after Hebbian learning. From Kim and Large (2021).

Up to this point, we have described single oscillations, as though behavior were governed by a single neural oscillator with a well-defined natural frequency that may adapt to input. For some experiments, this serves as a sufficient minimal model. However, in a more general framework, we may postulate multiple oscillatory circuits with a range of frequencies that interact within a network, as illustrated in Figure 11A. This more general framework is more physiologically realistic (Buzsáki and Draguhn, 2004; Buzsáki, 2006; Pittman-Polletta et al., 2021), and provides realistic predictions about EEG and MEG responses to complex rhythms, while making behavioral predictions about the perception of pulse and meter. However, modeling networks requires consideration of the roles of learning as well as neural time delays.

The single-oscillation model of Eq. 9 can be generalized to a network model by assuming a gradient of frequencies, which may include the delta and/or theta band frequencies as follows:


(10) $$\tau_{i}\,\frac{d\,z_{i}}{d\,t}=z_{i}\,\left(a_{i}+b_{i}\,{\left|z_{i}\right|}^{2}+\frac{d_{i}\,{\left|z_{i}\right|}^{4}}{1-{\left|z_{i}\right|}^{2}}\right)+\sum_{j\neq i}c_{i\,j}\,\frac{z_{j}}{1-z_{j}}\,\frac{1}{1-{\bar{z}}_{i}}$$

where τ_*i*_ = 2π/ω_*i*_ is the period of the ith oscillator, *a*_*i*_ = α_*i*_ + 2π*i*, *b*_*i*_ = β_1*i*_ + *i*δ_1*i*_, *d*_*i*_ = β_2*i*_ + *i*δ_2*i*_, and *c*_*ij*_ is a complex coefficient representing the strength and phase of the connection from the jth oscillator to the ith oscillator. In such a network, rather than individual oscillations adapting frequency over a wide range to match the stimulus frequency, the amplitude peaks within a network are determined by the stimulus frequencies. These networks can consist of E-I oscillators or a network of canonical oscillators, and the behaviors in the two models are comparable (see Figure 11B; Large et al., 2010). Thanks to mathematical tractability, the canonical model allows close analysis of nonlinear resonance in gradient frequency neural networks (Figure 11C; Kim and Large, 2019).

In neural mass models such as the Wilson-Cowan model, synaptic connections between oscillatory units determine their phase relations (Figure 11D). When synaptic connections are made plastic with Hebbian learning, neural mass models can learn and retain the phase relationships between frequency components in external signals (Hoppensteadt and Izhikevich, 1996b). In the canonical network model, connections within and between networks can be learned given a rule of the following form:


(11) $$\tau_{i\,j}\,\frac{d\,c_{i\,j}}{d\,t}=-\gamma_{i\,j}\,c_{i\,j}+\kappa_{i\,j}\,\frac{z_{i}}{1-z_{i}}\,\frac{{\bar{z}}_{j}}{1-{\bar{z}}_{j}}$$

where τ_*ij*_ is the time constant for learning, γ_*ij*_ is the decay rate, and κ_*ij*_ is the learning rate (Kim and Large, 2021). According to the multifrequency Hebbian learning rule, a connection strengthens (i.e., |*c*_*ij*_| increases) when connected oscillators are stably mode locked. In addition, the phase of the connection (*Arg c*_*ij*_) converges to the relative phase maintained between the oscillators. In this way, the network can learn and remember a multifrequency signal, such as a musical rhythm, as a pattern of nonlinear resonance among frequencies (Figure 11E). Finally, real neural networks also have time-delays, and an important goal for future research is to fully incorporate time delays into our models (see section “6.2.1. Transmission delay and negative mean asynchrony”).

In the next sections, we consider how to extend these models in light of the fact that multiple brain areas interact for rhythm processing, and we address the questions of how complex rhythms may be learned, produced, and coordinated.

## 6. Complex rhythms and multi-area modeling

A single heterogeneous-frequency oscillator network may be insufficient to explain complex rhythmic phenomena such as the ability to extract a pulse frequency not present in the input rhythm (Large et al., 2015). In this section we describe approaches to integrating multiple networks or brain areas to account for more complex aspects of rhythmic behavior.

### 6.1. Rhythm perception involves multiple brain areas

**FIGURE 12:**
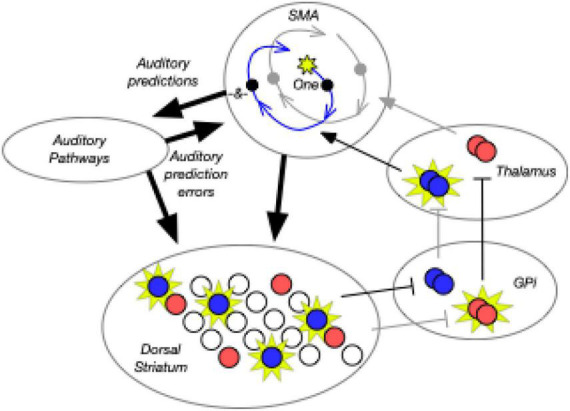
Basal ganglia sequences the beat-tracking trajectories in supplementary motor area (SMA). Neural firing rates in SMA follow a trajectory which tracks and anticipates progress through a beat cycle, informing auditory event predictions including the beat itself and other sounds locked to the cycle (such as a subdivision of the beat, indicated by the “&” symbol on the trajectory). The specifications of this trajectory, including tempo and subdivision, are determined by selective disinhibition by the basal ganglia via the “direct pathway”. A population specific to an intermediate tempo is active in dorsal striatum (blue), as opposed to one specific to a slower tempo (red). Acting through the internal segment of the globus pallidus (GPi), this population disinhibits a thalamic population, which provides SMA with excitation specific to that trajectory (blue) and not, for example, a slower one (gray). Auditory prediction errors act to update the phase of cyclical activity in SMA (phase correction) and update the active population in striatum (tempo correction).

As cited in the section “2. Neuroscience of rhythm processing,” imaging studies provide evidence for a number of areas that interact to predict upcoming sounds. Patel and Iversen (2014) proposed a phenomenological conceptual model (ASAP) that proposes that the motor system is integral to priming the sensory system to make timing judgments. More recently, Cannon and Patel (2021) suggested a neurophysiological implementation of the motor component of the ASAP scheme (Figure 12), perhaps analogous to the motor subcircuit of the Egger et al. (2020) model. They describe a recurrent neural network in Supplementary Motor Area (SMA) that entrains to auditory input at a beat level and that is embedded in a multi-area brain loop through supplementary motor area, thalamus, and basal ganglia that can serve to provide higher level metrical patterning and sequencing. This organization allows the SMA network to recognize and generate rhythm only at characteristic beat time scales, offloading higher-level structure to striatal networks known to be important for motor sequencing. Input to SMA along this pathway also may help SMA maintain a specific tempo through complex rhythms and during beat continuation.

### 6.2. Structure and perception in NRT network models

**FIGURE 13:**
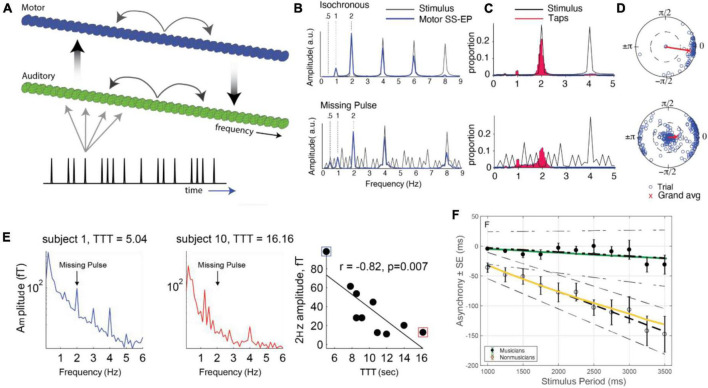
**(A)** A model of interacting oscillators in two reciprocally connected networks (auditory and motor). The auditory network can be driven with musical rhythmic stimuli, activating also the motor network due to the reciprocal connections. **(B)** The FFT of two rhythms (black line) and activation of the model’s motor network (blue line) when the model is driven by each: the top shows an isochronous rhythm 2 Hz and the bottom shows a “missing pulse” rhythm (i.e., a type of syncopated rhythm where there is no energy at the frequency of the perceived beat of 2 Hz). **(C)** Distributions of the tapping frequency of humans (red) while tapping with the isochronous or the “missing pulse” rhythm. **(D)** The phase of humans tapping with the isochronous or the “missing pulse” rhythm, computed at the trial (blue o’s) and “grand average” levels [red x’s; **(A–D)** adapted from Large et al. (2015)]. **(E)** The MEG spectrum of two individuals listening to “missing pulse” rhythms, one who was able to perceive the pulse of the syncopated rhythm faster (left, blue) than the other (center, red). The right panel shows correlation between the 2 Hz MEG amplitude and the time to start tapping (TTT) at 2 Hz [adapted from Tal et al. (2017)]. **(F)** NMA (means, and standard errors, and linear regressions) in musicians and non-musicians who tapped to an isochronous stimulus. Fits of an oscillator model with delayed feedback (Roman et al., 2019), for musicians (green) and non-musicians (yellow) [adapted from Roman et al. (2019)].

To account for empirical finding on the involvement of both motor and auditory networks, Large and collaborators (Large et al., 2015) have proposed a model that includes two heterogeneous-frequency networks, one for the dynamics of auditory cortex, and a second for motor cortex (e.g., SMA), and bidirectional coupling between the networks reflects learning of the lowest-order modes (2:1, 3:1, 1:2, and 1:3; see Eqs. 11, 12; Figure 13A). Amplitude dynamics of the canonical model are exploited to posit different parameter regimes for the two networks. Auditory dynamics operate near a Hopf bifurcation (Figures 10C, D; see e.g., Lakatos et al., 2013), making rhythmic responses in the auditory network essentially transient. Motor dynamics operate near a saddle-node bifurcation of periodic orbits (Figures 10E, F), so that each subpopulation can display transient response (fixed point dynamic) or a sustained response (limit cycle dynamics), with the two separated by a bottleneck threshold. Stimulation that is strong enough and long enough can trigger sustained motor network activity, similar to a pattern generator circuit (Kopell and Ermentrout, 1988; Marder and Bucher, 2001; Yuste et al., 2005; Guertin, 2009). Such a model could explain synchronization-continuation behavior, because a periodic auditory stimulus would trigger a sustained limit cycle in the motor network. It could also explain perception of musical rhythm, such that the stimulus triggers multiple, coupled limit cycles to embody the perception of metrical structure.

This model was tested using “missing-pulse” rhythms, in which the perception of pulse and meter *require* nonlinear responses (Large et al., 2015). Figure 13B shows the mean field response of auditory (bottom) and motor network (top) to a missing pulse rhythm. Frequency analysis of the acoustic stimulus shows no energy in the stimulus rhythm at 2 Hz – the hypothetical pulse frequency – or at 1 Hz – a subharmonic metrical frequency. Note that in these parameter regimes the auditory network rather faithfully reproduces the acoustic input, while the motor response contains a strong periodic component that corresponds to the hypothetical pulse of the input rhythm.

Experiments in human participants (Figures 13C, D) revealed that the hypothetical pulse (2 Hz) is perceived most often in these rhythms, and that pulse phase is bistable, also matching model predictions. Moreover, some subjects do not perceive a pulse in these highly syncopated rhythms (cf., Palmer et al., 2014). A follow-up MEG experiment verified that some subjects showed a strong neural response at the pulse frequency, while others did not (Figure 13E; Tal et al., 2017). Moreover, the amplitude of the 2 Hz neural responses matched performance in the pulse perception task, suggesting that the perception of pulse depends on the strength of the neural response. The model is also consistent with a series of experiments that have measured the steady-state evoked potential (SS-EP; see e.g., Nozaradan et al., 2011, 2012), showing enhancement of metrical frequencies in neural responses to music-like rhythms, and responses at frequencies not present in stimulus rhythms. Note that responses at the missing pulse frequency rule out passive, linear responses (i.e., ERP’s) because a linear response would similarly display a “missing pulse” frequency (see Large et al., 2015; Haegens and Zion Golumbic, 2018).

#### 6.2.1. Transmission delay and negative mean asynchrony

In considering the behavior of brain networks, it is important to also consider the existence of transmission delays. To our knowledge, no network models of musical rhythm have included such delays. However, one recent model has considered what the consequence of a transmission delay may be in a reciprocally connected network, using a single oscillator with delayed feedback as a simplified model.


(12) $$\frac{1}{f}\,\frac{d\,z}{d\,t}=z\,\left(\alpha +i\,2\,\pi +\beta \,{\left|z\right|}^{2}\right)+x\,\left(t\right)-\frac{d}{f}\,z\,\left(t-\tau \right)$$

Here *x*(*t*) is a unit-magnitude complex-valued sinusoid (or unit-magnitude square-wave) that captures the external stimulus, and the delayed feedback term has strength *d* and a delay of τ seconds. As a model of synchronization in the presence of delayed feedback, it is able to reproduce the tendency of taps to precede acoustic events, NMA (Repp, 2005). This type of anticipation is known as “strong anticipation,” since it results from the physical coupling of a system and its environment, and it does not depend on an internal representation of the stimulus (Dubois, 2001; Stepp and Turvey, 2010). This model provides an excellent fit to data from a detailed investigation of NMA (see Repp and Doggett, 2007; Roman et al., 2019). It reproduced the anticipatory tendencies empirically observed in musicians and nonmusicians for stimuli with different tempi (Figure 13F; see Repp and Doggett, 2007). It was also able to explain behavioral data from two turn-taking synchronization tasks between two humans with and without an additional acoustic transmission delay between participants (Chafe et al., 2010; Nowicki et al., 2013). Future models will be required to assess the effect of delays in more realistic networks.

Interestingly, the neuro-mechanistic model described above in the section “3. Models for time-keeping, beat generation and beat perception” (Bose et al., 2019) displays an intrinsic NMA, but not due to time-delay, rather due to its mechanism for measuring time intervals. Simulations of the Egger model (Egger et al., 2020) also exhibit a modest NMA, however the mechanism is not well-understood. It is not yet known whether either model is flexible enough to capture the empirical results (e.g., Repp and Doggett, 2007). PIPPET’s objective of aligning inferred phase with incoming events seems incompatible with this type of persistent synchronization error, although with the addition of motor output, it may ultimately prove compatible with the NMA findings.

## 7. Learning and development

The premature infant brain tracks beat and meter frequencies in auditory rhythms (Edalati et al., 2023), suggesting an innate characteristic of human neural activity. At the same time, rhythm processing is highly influenced by experience. Rhythms are experienced even before birth, for example, in the maternal heart beat, and through rhythmic movements, both of the mother but also from the fetus’s own arm and leg movements that have spring-like properties. After birth, infants become enculturated (specialized) at processing the beat and metrical structures in the music in their environment, and will even regularize rhythmic input to fit these metrical structures, suggesting that learning plays a large role in rhythm perception (Hannon and Trainor, 2007; Trainor and Marsh-Rollo, 2019). The PIPPET model proposes that rhythm perception develops through the refinement of patterns of temporal expectancy that act as generative models describing the probability of sound events at each phase of a meter or beat. The process of developing these expectations has not yet been modeled – instead, the model is “seeded” with one or more expectation patterns, which may represent any combination of learned and innate patterning with arbitrary temporal structure. NRT instead proposes that rhythm perception develops through learning of connections between circuits that are inherently oscillatory. This process allows learning through experience, but also explains why some rhythmic relationships (i.e., smaller integer ratios; cf., Kim and Large, 2019, 2021) are easier to learn.

The NRT framework incorporates learning as attunement of oscillatory networks to the rhythmic structure of the environment on multiple time-scales. When formalized in physiologically informed dynamical models based on Hebbian learning (Hoppensteadt and Izhikevich, 1996b; Kim and Large, 2021), this provides a theoretical framework for generating testable empirical predictions about musical rhythm development (Figure 13A; see Tichko et al., 2022). Building on the meter perception model above, this theoretical framework hypothesizes rhythmic attunement on (at least) three distinct timescales. On a timescale of seconds, people attune to the frequency and structure of ongoing musical rhythms (e.g., tempo adaptation; see Roman et al., 2023). On a timescale of months, children’s perceptual systems attune to culture-specific rhythmic structures (e.g., within-network connections; Tichko and Large, 2019). And at a timescale of years, children learn to flexibly coordinate movements with complex rhythms in culture-specific ways (between-network connections; Tichko et al., 2022).

**FIGURE 14:**
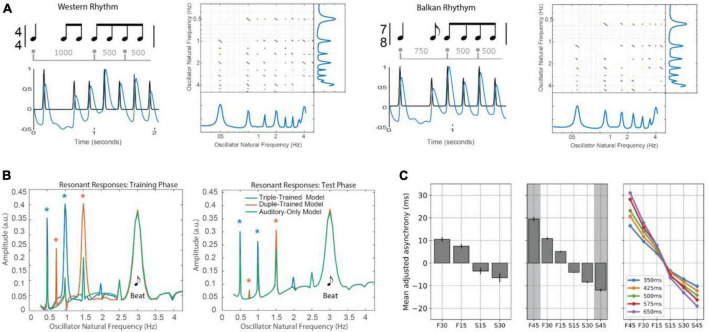
Learning and development. **(A)** Mean-field response of the untrained network to Western and Balkan rhythms (below each musical notation). Connection matrices and oscillator amplitudes after unsupervised learning (right from each music notation). From Tichko and Large (2019). **(B)** Resonant responses (i.e., average oscillatory activity) of models trained with either duple or triple auditory-vestibular stimulation (red and blue, respectively; duple- and triple-related frequencies are marked with asterisks) or auditory-only stimulation (green) during the final half of the training procedure (left). Resonant responses for the trained models during the final half of the test procedure with auditory-only stimulation (right). From Tichko et al. (2021). **(C)** Simulation and prediction of the mean adjusted asynchrony between a musician’s performance and a metronome beat for different tempi relative to the musician’s spontaneous motor tempo (SMT). F30: metronome period 30% shorter than the SMT, S15: metronome period 15% longer than the SMT, etc. Behavioral data from Scheurich et al. (2018) (left). Simulation results for a neural resonance model with elastic frequency learning, with predictions for 45% faster and slower conditions (middle). Model predictions for different natural (spontaneous) periods (right). From Roman et al. (2023).

As an example of perceptual learning, consider that a perceived beat need not be purely periodic. Non-isochronous meters (Brãiloiu, 1984; London, 2012) – which occur in musics from southeastern Europe, Mali, Turkey, and India (Clayton, 2008; Polak, 2010; Bates, 2011; Kirilov, 2015) – display categorically unequal durations between beats (Figure 14A). In learning the meter of musical rhythms, infants’ and children’s perceptual systems become attuned to culture-specific rhythmic patterns, displaying a kind of perceptual fine-tuning called perceptual narrowing (Hannon and Trehub, 2005). In one model, an untrained auditory network mean field veridically reproduced every rhythm, as in the model of Large et al. (2015). However, during unsupervised learning, the network learned connections between the oscillators activated by the training rhythms (Figure 14A). This affected the networks’ responses to violations of the metrical structure of native and nonnative rhythms, a pattern of findings that mirrored the behavioral data on infants’ perceptual narrowing to musical rhythms (Tichko and Large, 2019). Thus, the network attuned to the rhythmic structure of either Western or non-Western training rhythms through the self-organization of network connections within the auditory network. Another model demonstrated that diffuse (not frequency-specific) low amplitude connections between vestibular and auditory networks can bootstrap plasticity. In a seminal study, Phillips-Silver and Trainor (2005) showed that infants could be biased in their perception of a bistable rhythm (i.e., Figure 1A) by bouncing them according to the desired pulse. In the network model, during a training phase input from the vestibular network affected the response of the auditory network. During the test phase infants preferred the pulse that corresponded to the bouncing, and this behavior was captured by the model due to short term plasticity (Tichko et al., 2021; see Figure 14B).

At the faster time scale, an oscillator’s natural frequency can represent the endogenous rhythms that manifest as an individual’s spontaneous rates of action (Scheurich et al., 2018). Evidence suggests that an individual’s spontaneous rate constantly acts as a pulling force, and that anticipation and lagging can be explained by whether the spontaneous rate is slower or faster than the stimulus (Scheurich et al., 2018; Zamm et al., 2018). Recently, Roman et al. (2023) developed a neural resonance model with elastic frequency learning that can explain how musicians synchronize with a musical stimulus at a frequency different from their spontaneous rates of movement. The model’s fast frequency Hebbian learning allows the oscillator to match the stimulus frequency, but the elastic force pulls the learned frequency toward the system’s original natural frequency (Ermentrout, 1991; Savinov et al., 2021). This model was able to explain human behavioral data relating spontaneous rates with anticipatory asynchronies between human actions and different musical stimuli (Figure 14C; Scheurich et al., 2018). Moreover, the model explained a musician’s tendency to return to their spontaneous rate during a continuation task (after a brief period of synchronization with a stimulus), and the absolute asynchronies observed when pairs of individuals with different spontaneous rates synchronize with each other (Zamm et al., 2018).

## 8. Discussion

The ability of humans to track temporal patterns, extract or infer a hierarchy of regular beats, predict upcoming sensory events, adapt to temporal perturbations, adjust to tempo changes, and synchronize movements to rhythmic beats is impressive and while many questions have been addressed, many questions reman. Here we explored several basic modeling approaches to understanding musical rhythms: small biophysical or rate-based neuronal networks, probability-based Bayesian models, entrainment-based oscillator models, and heterogeneous frequency networks of neural oscillators with Hebbian learning. Neurophysiological measures show that intrinsic brain oscillations are ubiquitous over a range of frequencies (including delta, alpha, beta, gamma), suggesting that neural oscillations are fundamental to how the brain works. In the section “3. Models for time-keeping, beat generation and beat perception,” at a basic level we described two types of models: information processing and dynamical systems models in which a model’s phase and period variables adapt to synchronize with an external sound sequence. Neuro-mechanistic models, either based on the Hodgkin-Huxley formalism or on a population firing rate approach are designed to be real-time adaptive, with adjustable biophysical parameters that affect the internal beat generator’s period and/or phase to achieve matching with the stimulus. The proposed adjustment rules of each of these models provide both motivation, as well as challenges for how to formulate behavioral time scale plasticity in neuronal terms to instantiate these learning rules. With the exception of entrainment-based oscillator models, these models in their current forms, however, assume an isochronous temporal stimulus. The entrainment-based oscillator models, however, can capture the perception of an isochronous beat within a complex musical rhythm. In the section “4. Inference models of rhythm perception” we reviewed Bayesian approaches to rhythm perception, focusing on a recent model in which the phase, tempo, and meter of an underlying cycle is inferred from a rhythmic surface. The key feature of this approach is real-time progression and adaptation of a probability distribution (an internal template) for the phases at which events are expected. Rather than linking behavior to mechanism, Bayesian approaches link behavior to an inference problem that the behavior seems calibrated to solve. As a result, the Bayesian model of rhythm perception is open to multiple possible physical instantiations. In the section “5. Neural resonance theory,” we reviewed a specific dynamical systems approach involving Neural Resonance Theory (NRT) and the mathematical concept of entrainment. The model formulation is canonical, describing the generic properties of a periodically forced system that is capable of displaying oscillations. By analogy to the local canonical description of behavior near a Hopf bifurcation, one obtains a differential equation in which the terms in the vector field are in the form of an infinite series expansion that has been truncated. This local description provides properties of synchronization modes (e.g., *n:m* entrainment) and thereby predicts structural constraints in perception. The coefficients in the canonical form are available for fitting to match behavioral phenomena but are not directly interpretable as neuronal level parameters. However, they may be interpreted as physiological hypotheses about the parameter regimes of network oscillations. A parameterized network also provides predictions about the structure of local field potentials, as measured for example in the SS-EP. In the sections “6. Complex rhythms and multi-area modeling” and “7. Learning and development,” we explored modeling approaches to more complex aspects of rhythm processing, including interactions between brain areas and the effects of experience and learning.

Each of these approaches leaves open questions about physiological mechanisms and neural instantiation, including specifics about the interactions between or coordination across the levels of brain areas, circuits, and cellular and synaptic properties. The neuro-mechanistic models that we reviewed do not yet address questions such as how a beat can be perceived from a complex rhythmic surface that may contain syncopation or lack power at the beat frequency. Thus, it is of interest to ask whether the insights from higher-level modeling inform the formulation of biophysically based network models (such as Wilson-Cowan like firing rate models) capable of addressing similar issues for complex rhythms. Conversely, a solution may require integration across levels. For example, can a network with biophysically plausible units be shown to operate in parameter regimes that are consistent with Neural Resonance Theory and/or Bayesian formulations? At the cellular and synaptic level, what are candidate ionic and synaptic currents that allow a neural network to operate over the relevant frequency range of roughly 0.5–8 Hz? Constraints at each higher level of modeling may affect possible lower-level instantiations, and lower levels may themselves turn out to impose neuro-mechanistic constraints on rhythm perception. Thus, the prospect of creating a beat-tracking model on the level of neural mechanisms remains an exciting challenge.

Models of beat perception operate on a variety of different time scales that reflect the complicated nature of both the music that is being learned, but also, the complexities of our physiological system. Adaptable time-keeper and oscillator-based models operate on short time scales on the order of hundreds of milliseconds to seconds (e.g., Large and Jones, 1999; Bose et al., 2019; Egger et al., 2020). At this time-scale, they address real-time issues such as resynchronization to perturbations, tempo changes, and distractors. Models based on Neural Resonance Theory (NRT; Large et al., 2015; Roman et al., 2023) and a Bayesian approach (PIPPET; Cannon, 2021) can also operate on short time scales; the former to display entrainment to a given temporal sound sequence and the latter to match expectations and predictions to a known expectation template. At longer time-scales of minutes to years, Hebbian-type synaptic plasticity comes into play and underlies learning in NRT, where connection weights between oscillatory elements are formed to account for priming, perceptual narrowing, development of synchronization capabilities, and enculturation. In the Bayesian approach, expectation templates are formed on longer time-scales of months to years to account for musical enculturation.

### 8.1. Relationship between Bayesian and oscillator models

The Bayesian PIPPET algorithm for perceptual entrainment to rhythm takes the form of a hybrid continuous/discrete dynamical system that closely resembles a pulse-forced oscillator. Work is currently underway that will explore this relationship more fully. However, PIPPET, and more generally, the dynamic Bayesian modeling approach, is fundamentally different from oscillator-based modeling approaches in its commitment to the concept of representation, allowing it to interface more closely with the conceptual toolbox of cognitive psychology. Bayesian modeling speaks explicitly about “expectation” and “prediction,” considering these to be the fundamental goal of the nervous system. The model’s expectations and predictions are formulated relative to a preexisting or an assumed set of familiar rhythmic “templates” that depend on exposure. It further provides a general statistical framework within which to model the role of learning, development, and enculturation in entrainment to rhythm (Kaplan et al., 2022) and offers a mathematical language in which one can easily describe learned connections between rhythm and instrumental voice or pitch (e.g., Pazdera and Trainor, 2022).

Neural Resonance Theory, on the other hand, conceives of perception, attention, generation, and entrainment of oscillations as manifestations of the properties of oscillatory neural circuits. It eschews the traditional notion of a cognitive representation. A neural oscillation is not a representation of a musical rhythm; rather, it is an actual rhythm that operates in accordance with physical laws; thus, it should be considered a physical embodiment, not an abstract representation (see Tichko et al., 2022). This implies that predictions about perception, action, and cognition can and should be derived from dynamical analysis of neurophysiological oscillation. NRT adds two other generic features of neural physiology, neural plasticity and transmission delay, providing detailed fits to empirical data on perception, attention, generation, and synchronization of musical rhythms.

Real-time adaptable neuro-mechanistic models that operate based on error-correction and learning rules can be viewed as preliminary attempts to provide linkage between the behavioral level and plausible neural mechanisms (Bose et al., 2019; Egger et al., 2020). Doelling et al. (2022) observe that a neuro-mechanistic firing-rate oscillator model with an adaptive frequency can reproduce aspects of participant performance that seem to follow Bayesian principles (see also Egger et al., 2020). Indeed, what is interesting is how closely recent Bayesian models of musical rhythm resemble oscillator models. For example, each has a phase and frequency that adapt to an incoming stimulus. Further, Bayesian and canonical models both view temporal expectation as an important aspect of rhythm cognition (Large and Jones, 1999; Cannon, 2021). Heggli et al. (2019) take an oscillator-based dynamical systems approach to modeling dyadic entrainment, but speak in the language of the Bayesian Brain. However, Bayesian models and entrainment-based oscillator models do have important differences. Bayesian models do not constrain the range of rhythmic patterns that can be learned and entrained to. Oscillator models are naturally amenable to mode-locking in which small integer ratio relationships are exhibited over a wide range of the relevant parameter space. As a result, oscillatory network models predict that small integer ratio relationships are more stable in perception and attention, easier to coordinate in performance and synchronization, and easier to learn in development. Stability in turn predicts that smaller integer relationships may be more common among the musical traditions of the world (cf., Clayton, 2008; Polak, 2010; Bates, 2011; Kirilov, 2015).

### 8.2. Future developments

In describing the various models, we have focused on stimulus and behavioral timescales in the delta frequency band. The models do not explicitly address how rhythmic input also associates with higher frequency oscillations present in the neural response. Both β (15–30 Hz) and γ (>30 Hz) band rhythms have been observed in auditory cortex during musical beat perception (Snyder and Large, 2005; Zanto et al., 2005; Fujioka et al., 2009, 2012). Beta band (centered around 20 Hz) has been of particular interest in the auditory domain, with studies showing that presentation of a rhythmic input leads to periodic fluctuations in beta power that also phase align to the stimulus. Because beta frequencies are much faster than those of typical ERPs, and are not found in the stimulus itself, it is useful for isolating internal computational mechanisms. It has been shown that in ambiguous rhythms, where strong beats in the metrical hierarchy could be perceived to be in different places in the sequence, beta responses are largest in response to events perceived (or imagined) to be strong (Iversen et al., 2009). Beta analyses can also help determine whether neural oscillations reflect endogenous predictive processes. For example, in an isochronous sequence, beta power decreases after the onset of each beat and rebounds to reach maximum power around the expected time of the next beat. Fujioka et al. (2012) presented isochronous sequences of tones at different tempos and showed that the time course of beta decrease after each tone was similar across tempos, but the slope of the beta power rebound was shallower for slower tempos, suggesting a neural mechanism for prediction of the expected time of the next beat. Chang et al. (2019) showed on a trial-by-trial basis that the depth of beta modulation prior to a tone in an isochronous sequence predicted the size of ERP components following that tone that relate to prediction error. Further, phase-amplitude coupling between lower and higher frequencies is also commonly found (e.g., delta phase and beta power for auditory rhythms). Its function is still under debate, but it may represent coordination of different neural systems (e.g., Hyafil et al., 2015; Chang et al., 2019). For auditory rhythms, delta-beta coupling may be particularly important as beta frequencies are prominently associated with the motor system and delta frequencies are in the range in which humans perceive beats in music and language, so this coupling may reflect auditory-motor loops used for accomplishing auditory rhythmic timing. Although biophysical models of cross-frequency coupling have been proposed (e.g., Malerba and Kopell, 2013; Stanley et al., 2019), none have specifically addressed the role of cross-frequency coupling in musical rhythm, and this represents a challenge for future research. The development of neurophysiologically plausible models will need to take into account the different roles of oscillations at these different frequencies.

Another challenge that has not been systematically addressed is the phenomenon of negative mean asynchrony (NMA) and a mechanistic explanation for it. NMA has been found in simulations of some of the models reviewed in this paper (Bose et al., 2019; Roman et al., 2019; Egger et al., 2020). In the neuro-mechanistic models (Bose et al., 2019; Egger et al., 2020), the phase correction sought to produce zero phase difference. However, in both models, NMA is an emergent property. In Bose et al. (2019), NMA is attributed to the non-linear frequency response properties of the biophysical model together with an asymmetry in a phase-learning rule. The explanation of NMA in the Egger et al. (2020) model remains unanswered. Roman et al. (2019) explained the NMA observed in human empirical data as being the consequence of transmission delays. While they used a single oscillator with delayed feedback, future work could use a network-based description to simulate oscillatory activity in specific brain areas and the transmission delay between them. Furthermore, empirical evidence shows that even though NMA is observed when individuals tap with an isochronous metronome (Repp and Doggett, 2007), it shrinks or is absent when tapping the pulse of more complicated rhythms (Large et al., 2015). Compared to a metronome, spectral analysis reveals that complex rhythms may display little or no energy at the beat frequency, even if energy is prominent at integer-ratio frequencies (Large et al., 2015). Thus for a complex rhythm, the absence of NMA could result from enhanced oscillator resonance with a rhythm’s frequency components other than the beat, and interference between them.

## 9. Conclusion

Our dynamic experiences of musical rhythm, which can be observed in psychological, developmental, neurophysiological, and neuroimaging experiments, provide numerous challenges and questions for theorists. There is a wealth of behavioral data on perception, attention, and coordination of musical rhythms, and an abundance of EEG, MEG, and functional imaging data in humans. However, in regard to neurophysiological data there is a paucity of recordings at the cell and circuit levels due in large part to the lack of available experimental animal models; only a few individual animals have been shown capable of perceiving rhythmicity as in music. Hence, there is a great deal of evidence to guide modeling at the behavioral and brain macroscopic, multi-areal levels, but relatively few guideposts for development of neuro-mechanistic dynamical models of rhythmic perception at local network or areal-interaction level. We view these circumstances as providing creative opportunities for development of theory and models from multiple approaches and at multiple levels.

It seems clear that behavioral models of musical rhythm must account for temporal expectancy, adaptation, and learning, as Bayesian models do. Bayesian models eschew neural mechanisms and instead work to describe rhythm perception in terms of general principles of perception and cognition. In doing so, they also account for behavioral phenomena such as expectation-related time distortion and the experience of groove by showing that they follow from these first principles.

Oscillator models that emphasize resonance and mode-locking also capture temporal expectancy. Additionally these types of models can account for structural constraints in perception, attention, and coordination. By considering physiological mechanisms of which we can be fairly certain such as neural oscillations, Hebbian plasticity, and neural transmission delays, such models have been used to account for an array of behavioral, developmental, and neurophysiological data on musical rhythm. These generic aspects of neurophysiology, focused on musical rhythm using the techniques of dynamical systems analysis, provides a powerful theoretical framework, and makes additional predictions about perceptual structures, neurophysiological responses, and developmental milestones, which have only begun to be tested.

But important questions remain open. At the level of neural systems, how can we account for the many brain areas that are activated by rhythmic stimuli and rhythmic tasks? Should we think in terms of interaction of oscillatory networks in multiple brain areas? Or do the excitatory and inhibitory populations – whose interactions generate oscillations – reside in different areas? Could some areas provide signals for controlling the parameters of oscillations, turning them on and off or controlling frequency? Perhaps some circuits oscillate, while others measure rhythmic time intervals. At the level of detailed neural circuits, relatively little is known that is directly relevant to this type of human behavior. Since the constraints are relatively modest, future researchers may explore plausible neuronal and circuit level mechanisms that can help reveal elemental neural processes and make additional behavioral predictions. Detailed knowledge about specific circuits and systems will be invaluable in developing clinical applications.

We are at the early stages of understanding rhythm perception at a detailed neural level and the roadway is wide. Here, we have described some basic phenomena of rhythm pattern perception and approaches to modeling them in an attempt to discern essential dynamical mechanisms. Although it seems indisputable that neural oscillations are involved in musical rhythm perception, we seek to be able to distinguish between or identify overlap among viable formulations that adapt in real-time and learn over longer time-scales. We hope this review tempts the reader to join in pursuing the formulation and development of models that can help address the many interesting questions about rhythm perception.

## Author contributions

All authors listed have made a substantial, direct, and intellectual contribution to the work, and approved it for publication.

## Figures and Tables

**Table:** 

**Term**	**Definition**
Rhythm	Time series with temporal predictability
Rhythmic surface; rhythmic pattern	Time series of sounds and silences in an auditory pattern
Beat	A sequence of perceived events (often periodic) that is implicit in a rhythm pattern
Metrical hierarchy (or “metrical structure” or meter)	Hierarchy of beats in which n successive beats are grouped at one level, forming a single (down-) beat at a higher level
Pulse	The main beat; the beat level a listener would tap when synchronizing with a rhythm
Tempo (or “musical tempo”)	The beat frequency, in beats per minute (bpm), or beats per second (bps, or Hz)
Isochronous sequence	A sequence of events that are evenly spaced in time
Synchronization-continuation	Synchronizing with a repetitive pattern and continuing production (perceptual or finger tapping) after stimulus removal
Negative mean asynchrony (NMA)	The tendency for a person’s movements to anticipate rhythmic events in synchronization tasks (e.g., tapping to the pulse); calculated by averaging the differences between the agent’s timing and the true stimulus timing.
Syncopation	A type of rhythmic complexity in which events tend not to coincide with the musical pulse
Entrainment	The response of an ongoing, self-sustained oscillation to an external periodic force resulting in temporal alignment between the two
Bayesian brain hypothesis	Framework that sees the brain as a machine that computes statistical properties of past events to optimize beliefs, expectations, and behavior for the future
Generative model	Uses Bayes rule to estimate the probability of a hidden state (*h*) given an observation (*o*). *p*(*h*| *o*) = *p*(*o*| *h*)*p*(*h*)/*p*(*o*)
Expectation template	Describes the temporal structure of the observer’s event expectations, and the degree to which an event is anticipated at time *t*
Canonical model (normal form)	A simple mathematical form that captures the common properties of a family of more detailed models that share certain local dynamics (such as being near a certain type of bifurcation)
Resonance	A general concept encompassing both passive resonance of linear systems and active resonance of nonlinear systems. Linear systems show resonant responses only at stimulus frequency (phase-locking), while nonlinear systems also resonate at integer ratios and combination frequencies (mode-locking).
Mode-locking	Phase alignment between different frequencies in which *k* cycles of one oscillation consistently align with *m* cycles of another oscillation (called *k*:*m* mode-locking)
Hebbian learning	Mechanism to update connections between neurons in a network, in response to a stimulus or change in intrinsic network activity
Transmission delay	A time delay in communication between neural areas, biophysically explained by long-range axonal transmission of information

## References

[B1] Arnal L. H. Giraud A.-L. (2012). Cortical oscillations and sensory predictions. *Trends Cogn. Sci.* 16 390–398. 10.1016/j.tics.2012.05.003 22682813

[B2] Auksztulewicz R. Myers N. E. Schnupp J. W. Nobre A. C. (2019). Rhythmic temporal expectation boosts neural activity by increasing neural gain. *J. Neurosci.* 39 9806–9817. 10.1523/jneurosci.0925-19.2019 31662425 PMC6891052

[B3] Barnes R. Jones M. R. (2000). Expectancy, attention, and time. *Cogn. Psychol.* 41 254–311. 10.1006/cogp.2000.0738 11032658

[B4] Bates V. C. (2011). Preparing rural music teachers: reflecting on “shared visions”. *J. Music Teach. Educ.* 20 89–98. 10.1177/1057083710377722

[B5] Bendixen A. Schroger E. Winkler I. (2009). I heard that coming: event-related potential evidence for stimulus-driven prediction in the auditory system. *J. Neurosci.* 29 8447–8451. 10.1523/jneurosci.1493-09.2009 19571135 PMC6665649

[B6] Bengtsson S. L. Ullén F. Ehrsson H. H. Hashimoto T. Kito T. Naito E. (2009). Listening to rhythms activates motor and premotor cortices. *Cortex* 45 62–71. 10.1016/j.cortex.2008.07.002 19041965

[B7] Betancourt A. Pérez O. Gámez J. Mendoza G. Merchant H. (2022). Amodal population clock in the primate medial premotor system for rhythmic tapping. *bioRxiv* [Preprint]. 10.1101/2022.08.14.503904 37838944

[B8] Bose A. Byrne Á Rinzel J. (2019). A neuromechanistic model for rhythmic beat generation. *PLoS Comput. Biol.* 15:e1006450. 10.1371/journal.pcbi.1006450 31071078 PMC6508617

[B9] Bouwer F. L. Van Zuijen T. L. Honing H. (2014). Beat processing is pre-attentive for metrically simple rhythms with clear accents: an ERP study. *PLoS One* 9:e97467. 10.1371/journal.pone.0097467 24870123 PMC4037171

[B10] Brãiloiu C. (1984). *Problems of ethnomusicology.* Cambridge: Cambridge University Press.

[B11] Breakspear M. (2017). Dynamic models of large-scale brain activity. *Nat. Neurosci.* 20 340–352. 10.1038/nn.4497 28230845

[B12] Bucy R. S. Joseph P. D. (2005). *Filtering for stochastic processes with applications to guidance*, 2nd Edn. Providence, RI: AMS Chelsea Pub.

[B13] Burger B. Thompson M. R. Luck G. Saarikallio S. H. Toiviainen P. (2014). Hunting for the beat in the body: on period and phase locking in music-induced movement. *Front. Hum. Neurosci.* 8:903. 10.3389/fnhum.2014.00903 25426051 PMC4224089

[B14] Buzsáki G. (2004). Large-scale recording of neuronal ensembles. *Nat. Neurosci.* 7 446–451. 10.1038/nn1233 15114356

[B15] Buzsáki G. (2006). *Rhythms of the brain.* Oxford: Oxford University Press. 10.1093/acprof:oso/9780195301069.001.0001

[B16] Buzsáki G. Draguhn A. (2004). Neuronal oscillations in cortical networks. *Science* 304 1926–1929. 10.1126/science.1099745 15218136

[B17] Byrne Á Rinzel J. Bose A. (2020). Order-indeterminant event-based maps for learning a beat. *Chaos* 30 083138. 10.1063/5.0013771 32872826

[B18] Cannon J. (2021). Expectancy-based rhythmic entrainment as continuous Bayesian inference. *PLoS Comput. Biol.* 17:e1009025. 10.1371/journal.pcbi.1009025 34106918 PMC8216548

[B19] Cannon J. Patel A. D. (2021). How beat perception co-opts motor neurophysiology. *Trends Cogn. Sci.* 25 137–150. 10.1016/j.tics.2020.11.002 33353800 PMC9440376

[B20] Carbajal G. V. Malmierca M. S. (2018). The neuronal basis of predictive coding along the auditory pathway: from the subcortical roots to cortical deviance detection. *Trends Hear.* 22:233121651878482. 10.1177/2331216518784822 30022729 PMC6053868

[B21] Cariani P. (2002). Temporal codes, timing nets, and music perception. 2002. *J. New Music Res.* 30 107–136.

[B22] Chafe C. Cáceres J.-P. Gurevich M. (2010). Effect of temporal separation on synchronization in rhythmic performance. *Perception* 39 982–992. 10.1068/p6465 20842974

[B23] Chang A. Bosnyak D. J. Trainor L. J. (2019). Rhythmicity facilitates pitch discrimination: differential roles of low and high frequency neural oscillations. *Neuroimage* 198 31–43. 10.1016/j.neuroimage.2019.05.007 31059798

[B24] Chang A. Livingstone S. R. Bosnyak D. J. Trainor L. J. (2017). Body sway reflects leadership in joint music performance. *Proc. Natl. Acad. Sci. U.S.A.* 114 E4134–E4141. 10.1073/pnas.1617657114 28484007 PMC5448222

[B25] Chen J. L. Penhune V. B. Zatorre R. J. (2008a). Listening to musical rhythms recruits motor regions of the brain. *Cereb. Cortex* 18 2844–2854. 10.1093/cercor/bhn042 18388350

[B26] Chen J. L. Penhune V. B. Zatorre R. J. (2008b). Moving on time: brain network for auditory-motor synchronization is modulated by rhythm complexity and musical training. *J. Cogn. Neurosci.* 20 226–239. 10.1162/jocn.2008.20018 18275331

[B27] Chen J. L. Zatorre R. J. Penhune V. B. (2006). Interactions between auditory and dorsal premotor cortex during synchronization to musical rhythms. *Neuroimage* 32 1771–1781. 10.1016/j.neuroimage.2006.04.207 16777432

[B28] Chen Y. Repp B. H. Patel A. D. (2002). Spectral decomposition of variability in synchronization and continuation tapping: comparisons between auditory and visual pacing and feedback conditions. *Hum. Movement Sci.* 21 515–532. 10.1016/s0167-9457(02)00138-0 12450682

[B29] Church R. M. Gibbon J. (1982). Temporal generalization. *J. Exp. Psychol.* 8 165–186. 10.1037/0097-7403.8.2.165 7069377

[B30] Cirelli L. K. Einarson K. M. Trainor L. J. (2014). Interpersonal synchrony increases prosocial behavior in infants. *Dev. Sci.* 17 1003–1011. 10.1111/desc.12193 25513669

[B31] Ciszak M. Marino F. Toral R. Balle S. (2004). Dynamical mechanism of anticipating synchronization in excitable systems. *Phys. Rev. Lett.* 93 114102. 10.1103/physrevlett.93.114102 15447342

[B32] Clayton M. (2008). *Time in Indian music: rhythm, metre, and form in North Indian rag performance.* Oxford: Oxford University Press.

[B33] Correa Á Nobre A. C. (2008). Neural modulation by regularity and passage of time. *J. Neurophysiol.* 100 1649–1655. 10.1152/jn.90656.2008 18632896

[B34] Crowe D. A. Zarco W. Bartolo R. Merchant H. (2014). Dynamic representation of the temporal and sequential structure of rhythmic movements in the primate medial premotor cortex. *J. Neurosci.* 34 11972–11983. 10.1523/jneurosci.2177-14.2014 25186744 PMC6608467

[B35] Dauer T. Nerness B. Fujioka T. (2020). Predictability of higher-order temporal structure of musical stimuli is associated with auditory evoked response. *Int. J. Psychophysiol.* 153 53–64. 10.1016/j.ijpsycho.2020.04.002 32325078

[B36] deGuzman G. C. Kelso J. A. S. (1991). Multifrequency behavioral patterns and the phase attractive circle map. *Biol. Cybern.* 64 485–495. 10.1007/bf00202613 1863660

[B37] Doelling K. B. Assaneo M. F. (2021). Neural oscillations are a start toward understanding brain activity rather than the end. *PLoS Biol.* 19:e3001234. 10.1371/journal.pbio.3001234 33945528 PMC8121326

[B38] Doelling K. B. Arnal L. H. Assaneo M. F. (2022). Adaptive oscillators provide a hard-coded Bayesian mechanism for rhythmic inference. *bioRxiv* [Preprint]. 10.1101/2022.06.18.496664

[B39] Doelling K. B. Assaneo M. F. Bevilacqua D. Pesaran B. Poeppel D. (2019). An oscillator model better predicts cortical entrainment to music. *Proc. Natl. Acad. Sci. U.S.A.* 116 10113–10121. 10.1073/pnas.1816414116 31019082 PMC6525506

[B40] Dotov D. Delasanta L. Cameron D. J. Large E. W. Trainor L. (2022). Collective dynamics support group drumming, reduce variability, and stabilize tempo drift. *eLife* 11:e74816. 10.7554/elife.74816 36317963 PMC9678363

[B41] Dubois D. M. (2001). Incursive and hyperincursive systems, fractal machine and anticipatory logic. *AIP Conf. Proc.* 573 437–451. 10.1063/1.1388710

[B42] Edalati M. Wallois F. Safaie J. Ghostine G. Kongolo G. Trainor L. J. (2023). Rhythm in the premature neonate brain: very early processing of auditory beat and meter. *J. Neurosci.* 43 2794–2802. 10.1523/jneurosci.1100-22.2023 36914264 PMC10089239

[B43] Egger S. W. Le N. M. Jazayeri M. (2020). A neural circuit model for human sensorimotor timing. *Nat. Commun.* 11:3933. 10.1038/s41467-020-16999-8 32770038 PMC7414125

[B44] Eguíluz V. M. Ospeck M. Choe Y. Hudspeth A. J. Magnasco M. O. (2000). Essential nonlinearities in hearing. *Phys. Rev. Lett.* 84 5232–5235. 10.1103/physrevlett.84.5232 10990910

[B45] Ellamil M. Berson J. Wong J. Buckley L. Margulies D. S. (2016). One in the dance: musical correlates of group synchrony in a real-world club environment. *PLoS One* 11:e0164783. 10.1371/journal.pone.0164783 27764167 PMC5072606

[B46] Elliott M. T. Wing A. M. Welchman A. E. (2014). Moving in time: Bayesian causal inference explains movement coordination to auditory beats. *Proc. R. Soc. B Biol. Sci.* 281:20140751. 10.1098/rspb.2014.0751 24850915 PMC4046422

[B47] Ermentrout B. (1991). An adaptive model for synchrony in the firefly Pteroptyx malaccae. *J. Math. Biol.* 29 571–585. 10.1007/bf00164052

[B48] Ermentrout B. (1998). Neural networks as spatio-temporal pattern-forming systems. *Rep. Progr. Phys.* 61 353–430. 10.1088/0034-4885/61/4/002

[B49] Ermentrout G. B. Cowan J. D. (1979). A mathematical theory of visual hallucination patterns. *Biol. Cybern.* 34 137–150. 10.1007/bf00336965 486593

[B50] Essens P. J. Povel D.-J. (1985). Metrical and nonmetrical representations of temporal patterns. *Percept. Psychophys.* 37 1–7. 10.3758/bf03207132 3991313

[B51] Fiveash A. Bedoin N. Gordon R. L. Tillmann B. (2021). Processing rhythm in speech and music: shared mechanisms and implications for developmental speech and language disorders. *Neuropsychology* 35 771–791. 10.1037/neu0000766 34435803 PMC8595576

[B52] Flaten E. Marshall S. A. Dittrich A. Trainor L. J. (2022). Evidence for top-down metre perception in infancy as shown by primed neural responses to an ambiguous rhythm. *Eur. J. Neurosci.* 55 2003–2023. 10.1111/ejn.15671 35445451

[B53] Friston K. (2005). A theory of cortical responses. *Philos. Trans. R. Soc. B Biol. Sci.* 360 815–836. 10.1098/rstb.2005.1622 15937014 PMC1569488

[B54] Friston K. (2010). The free-energy principle: a unified brain theory? *Nat. Rev. Neurosci.* 11 127–138. 10.1038/nrn2787 20068583

[B55] Fujioka T. Trainor L. J. Large E. W. Ross B. (2009). Beta and gamma rhythms in human auditory cortex during musical beat processing. *Ann. N.Y. Acad. Sci.* 1169 89–92. 10.1111/j.1749-6632.2009.04779.x 19673759

[B56] Fujioka T. Trainor L. J. Large E. W. Ross B. (2012). Internalized timing of isochronous sounds is represented in neuromagnetic beta oscillations. *J. Neurosci.* 32 1791–1802. 10.1523/jneurosci.4107-11.2012 22302818 PMC6703342

[B57] Gibbon J. Church R. M. Meck W. H. (1984). Scalar timing in memory. *Ann. N.Y. Acad. Sci.* 423 52–77. 10.1111/j.1749-6632.1984.tb23417.x 6588812

[B58] Glass L. (2001). Synchronization and rhythmic processes in physiology. *Nature* 410 277–284. 10.1038/35065745 11258383

[B59] Glass L. Mackey M. C. (1988). *From clocks to chaos: the rhythms of life.* Princeton, NJ: Princeton University Press, 10.1515/9780691221793

[B60] Grahn J. A. Brett M. (2007). Rhythm and beat perception in motor areas of the brain. *J. Cogn. Neurosci.* 19 893–906. 10.1162/jocn.2007.19.5.893 17488212

[B61] Grahn J. A. Brett M. (2009). Impairment of beat-based rhythm discrimination in Parkinson’s disease. *Cortex* 45 54–61. 10.1016/j.cortex.2008.01.005 19027895

[B62] Grahn J. A. Rowe J. B. (2009). Feeling the beat: premotor and striatal interactions in musicians and nonmusicians during beat perception. *J. Neurosci.* 29 7540–7548. 10.1523/jneurosci.2018-08.2009 19515922 PMC2702750

[B63] Grahn J. A. Rowe J. B. (2013). Finding and feeling the musical beat: striatal dissociations between detection and prediction of regularity. *Cereb. Cortex* 23 913–921. 10.1093/cercor/bhs083 22499797 PMC3593578

[B64] Grube M. Cooper F. E. Chinnery P. F. Griffiths T. D. (2010). Dissociation of duration-based and beat-based auditory timing in cerebellar degeneration. *Proc. Natl. Acad. Sci. U.S.A.* 107 11597–11601. 10.1073/pnas.0910473107 20534501 PMC2895141

[B65] Guertin P. A. (2009). The mammalian central pattern generator for locomotion. *Brain Res. Rev.* 62 45–56. 10.1016/j.brainresrev.2009.08.002 19720083

[B66] Haegens S. Zion Golumbic E. (2018). Rhythmic facilitation of sensory processing: a critical review. *Neurosci. Biobehav. Rev.* 86 150–165. 10.1016/j.neubiorev.2017.12.002 29223770

[B67] Haken H. Kelso J. A. S. Bunz H. (1985). A theoretical model of phase transitions in human hand movements. *Biol. Cybern.* 51 347–356. 10.1007/bf00336922 3978150

[B68] Hannon E. E. Trainor L. J. (2007). Music acquisition: effects of enculturation and formal training on development. *Trends Cogn. Sci.* 11 466–472. 10.1016/j.tics.2007.08.008 17981074

[B69] Hannon E. E. Trehub S. E. (2005). Tuning in to musical rhythms: infants learn more readily than adults. *Proc. Natl. Acad. Sci. U.S.A.* 102 12639–12643. 10.1073/pnas.0504254102 16105946 PMC1194930

[B70] Hansen N. C. Kragness H. E. Vuust P. Trainor L. Pearce M. T. (2021). Predictive uncertainty underlies auditory boundary perception. *Psychol. Sci.* 32 1416–1425. 10.1177/0956797621997349 34409898

[B71] Hary D. Moore G. P. (1987). Synchronizing human movement with an external clock source. *Biol. Cybern.* 56 305–311. 10.1007/bf00319511 3620530

[B72] Heggli O. A. Cabral J. Konvalinka I. Vuust P. Kringelbach M. L. (2019). A Kuramoto model of self-other integration across interpersonal synchronization strategies. *PLoS Comput. Biol.* 15:e1007422. 10.1371/journal.pcbi.1007422 31618261 PMC6816575

[B73] Heggli O. A. Konvalinka I. Kringelbach M. L. Vuust P. (2021). A metastable attractor model of self-other integration (MEAMSO) in rhythmic synchronization. *Philos. Trans. R. Soc. B Biol. Sci.* 376:20200332. 10.1098/rstb.2020.0332 34420393 PMC8380980

[B74] Henry M. J. Herrmann B. (2014). Low-frequency neural oscillations support dynamic attending in temporal context. *Timing Time Percept.* 2 62–86. 10.1163/22134468-00002011

[B75] Henry M. J. Herrmann B. Obleser J. (2014). Entrained neural oscillations in multiple frequency bands comodulate behavior. *Proc. Natl. Acad. Sci. U.S.A.* 111 14935–14940. 10.1073/pnas.1408741111 25267634 PMC4205645

[B76] Herbst S. K. Obleser J. (2019). Implicit temporal predictability enhances pitch discrimination sensitivity and biases the phase of delta oscillations in auditory cortex. *Neuroimage* 203 116198. 10.1016/j.neuroimage.2019.116198 31539590

[B77] Hickok G. Farahbod H. Saberi K. (2015). The rhythm of perception. *Psychol. Sci.* 26 1006–1013. 10.1177/0956797615576533 25968248 PMC4504793

[B78] Hodgkin A. L. Huxley A. F. (1952). A quantitative description of membrane current and its application to conduction and excitation in nerve. *J. Physiol.* 117 500–544. 10.1113/jphysiol.1952.sp004764 12991237 PMC1392413

[B79] Hoppensteadt F. C. Izhikevich E. M. (1996a). Synaptic organizations and dynamical properties of weakly connected neural oscillators I. analysis of a canonical model. *Biol. Cybern.* 75 117–127. 10.1007/s004220050279 8855350

[B80] Hoppensteadt F. C. Izhikevich E. M. (1996b). Synaptic organizations and dynamical properties of weakly connected neural oscillators II. learning phase information. *Biol. Cybern.* 75 129–135. 10.1007/s004220050280 8855351

[B81] Hoppensteadt F. C. Izhikevich E. M. (1997). *Weakly connected neural networks.* Berlin: Springer. 10.1007/978-1-4612-1828-9

[B82] Hove M. J. Risen J. L. (2009). It’s all in the timing: interpersonal synchrony increases affiliation. *Soc. Cogn.* 27 949–960. 10.1521/soco.2009.27.6.949

[B83] Huron D. (2008). *Sweet anticipation: music and the psychology of expectation.* Cambridge, MA: MIT Press.

[B84] Hyafil A. Giraud A.-L. Fontolan L. Gutkin B. (2015). Neural cross-frequency coupling: connecting architectures, mechanisms, and functions. *Trends Neurosci.* 38 725–740. 10.1016/j.tins.2015.09.001 26549886

[B85] Iversen J. R. Repp B. H. Patel A. D. (2009). Top-down control of rhythm perception modulates early auditory responses. *Ann. N.Y. Acad. Sci.* 1169 58–73. 10.1111/j.1749-6632.2009.04579.x 19673755

[B86] Izhikevich E. M. (2007). *Dynamical systems in neuroscience.* Cambridge, MA: MIT Press.

[B87] Jacoby N. McDermott J. H. (2017). Integer ratio priors on musical rhythm revealed cross-culturally by iterated reproduction. *Curr. Biol.* 27 359–370. 10.1016/j.cub.2016.12.031 28065607

[B88] Jacoby N. Polak R. Grahn J. Cameron D. J. Lee K. M. Godoy R. (2021). Universality and cross-cultural variation in mental representations of music revealed by global comparison of rhythm priors. *PsyArXiv* [Preprint]. 10.31234/osf.io/b879v PMC11132990 38438653

[B89] Jagacinski R. J. Peper C. L. E. Beek P. J. (2000). Dynamic, stochastic, and topological aspects of polyrhythmic performance. *J. Motor Behav.* 32 323–336. 10.1080/00222890009601383 11114226

[B90] Jones M. R. (1976). Time, our lost dimension: toward a new theory of perception, attention, and memory. *Psychol. Rev.* 83 323–355. 10.1037/0033-295x.83.5.323 794904

[B91] Jones M. R. Boltz M. (1989). Dynamic attending and responses to time. *Psychol. Rev.* 96 459–491. 10.1037/0033-295x.96.3.459 2756068

[B92] Jones M. R. Johnston H. M. Puente J. (2006). Effects of auditory pattern structure on anticipatory and reactive attending. *Cogn. Psychol.* 53 59–96. 10.1016/j.cogpsych.2006.01.003 16563367

[B93] Jones M. R. Moynihan H. MacKenzie N. Puente J. (2002). Temporal aspects of stimulus-driven attending in dynamic arrays. *Psychol. Sci.* 13 313–319. 10.1111/1467-9280.00458 12137133

[B94] Kaplan T. Cannon J. Jamone L. Pearce M. (2022). Modeling enculturated bias in entrainment to rhythmic patterns. *PLoS Comput. Biol.* 18:e1010579. 10.1371/journal.pcbi.1010579 36174063 PMC9553061

[B95] Kasdan A. V. Burgess A. N. Pizzagalli F. Scartozzi A. Chern A. Kotz S. A. (2022). Identifying a brain network for musical rhythm: a functional neuroimaging meta-analysis and systematic review. *Neurosci. Biobehav. Rev.* 136:104588. 10.1016/j.neubiorev.2022.104588 35259422 PMC9195154

[B96] Keller P. E. Repp B. H. (2005). Staying offbeat: sensorimotor syncopation with structured and unstructured auditory sequences. *Psychol. Res.* 69 292–309. 10.1007/s00426-004-0182-9 15616863

[B97] Kelso J. A. S. (1995). *Dynamic patterns: the self-organization of brain and behavior.* Cambridge, MA: MIT Press.

[B98] Kelso J. A. S. (2000). “Principles of dynamic pattern formation and change for a science of human behavior,” in *Developmental science and the holistic approach*, eds Bergman L. R. Cairns R. B. Nilsson L.-G. Nystedt L. (New York: Routledge).

[B99] Kim J. C. Large E. W. (2015). Signal processing in periodically forced gradient frequency neural networks. *Front. Comput. Neurosci.* 9:152. 10.3389/fncom.2015.00152 26733858 PMC4689852

[B100] Kim J. C. Large E. W. (2019). Mode locking in periodically forced gradient frequency neural networks. *Phys. Rev. E* 99 022421. 10.1103/physreve.99.022421 30934299

[B101] Kim J. C. Large E. W. (2021). Multifrequency Hebbian plasticity in coupled neural oscillators. *Biol. Cybern.* 115 43–57. 10.1007/s00422-020-00854-6 33399947

[B102] Kirilov K. S. (2015). *Bulgarian harmony.* Milton Park: Routledge. 10.4324/9781315261126

[B103] Koelsch S. Vuust P. Friston K. (2019). Predictive processes and the peculiar case of music. *Trends Cogn. Sci.* 23 63–77. 10.1016/j.tics.2018.10.006 30471869

[B104] Konvalinka I. Vuust P. Roepstorff A. Frith C. D. (2010). Follow you, follow me: continuous mutual prediction and adaptation in joint tapping. *Q. J. Exp. Psychol.* 63 2220–2230. 10.1080/17470218.2010.497843 20694920

[B105] Kopell N. Ermentrout G. B. (1988). Coupled oscillators and the design of central pattern generators. *Math. Biosci.* 90 87–109. 10.1016/0025-5564(88)90059-4

[B106] Lakatos P. Barczak A. Neymotin S. A. McGinnis T. Ross D. Javitt D. C. (2016). Global dynamics of selective attention and its lapses in primary auditory cortex. *Nat. Neurosci.* 19 1707–1717. 10.1038/nn.4386 27618311 PMC5127770

[B107] Lakatos P. Gross J. Thut G. (2019). A new unifying account of the roles of neuronal entrainment. *Curr. Biol.* 29 R890–R905. 10.1016/j.cub.2019.07.075 31550478 PMC6769420

[B108] Lakatos P. Karmos G. Mehta A. D. Ulbert I. Schroeder C. E. (2008). Entrainment of neuronal oscillations as a mechanism of attentional selection. *Science* 320 110–113. 10.1126/science.1154735 18388295

[B109] Lakatos P. Musacchia G. O’Connel M. N. Falchier A. Y. Javitt D. C. Schroeder C. E. (2013). The spectrotemporal filter mechanism of auditory selective attention. *Neuron* 77 750–761. 10.1016/j.neuron.2012.11.034 23439126 PMC3583016

[B110] Lambert A. J. Weyde T. Armstrong N. (2016). “Adaptive frequency neural networks for dynamic pulse and metre perception,” in *Proceedings of the 17th international society for music information retrieval conference*, eds Mandel M. I. Devaney J. Turnbull D. Tzanetakis G. (New York, NY), 60–66.

[B111] Large E. W. (2008). “Resonating to musical rhythm: theory and experiment,” in *The psychology of time*, ed. Grondin S. (Bingley: Emerald), 189–231. 10.1068/i0665

[B112] Large E. W. Almonte F. V. Velasco M. J. (2010). A canonical model for gradient frequency neural networks. *Phys. D Nonlinear Phenomena* 239 905–911. 10.1016/j.physd.2009.11.015

[B113] Large E. W. Jones M. R. (1999). The dynamics of attending: how people track time-varying events. *Psychol. Rev.* 106 119–159. 10.1037/0033-295x.106.1.119

[B114] Large E. W. Kolen J. F. (1994). Resonance and the perception of musical meter. *Connect. Sci.* 6 177–208. 10.1080/09540099408915723

[B115] Large E. W. Palmer C. (2002). Perceiving temporal regularity in music. *Cogn. Sci.* 26 1–37. 10.1207/s15516709cog2601_1

[B116] Large E. W. Snyder J. S. (2009). Pulse and meter as neural resonance. *Ann. N.Y. Acad. Sci.* 1169 46–57. 10.1111/j.1749-6632.2009.04550.x 19673754

[B117] Large E. W. Fink P. Kelso S. J. (2002). Tracking simple and complex sequences. *Psychol. Res.* 66 3–17. 10.1007/s004260100069 11963276

[B118] Large E. W. Herrera J. A. Velasco M. J. (2015). Neural networks for beat perception in musical rhythm. *Front. Syst. Neurosci.* 9:159. 10.3389/fnsys.2015.00159 26635549 PMC4658578

[B119] Lense M. D. Ladányi E. Rabinowitch T.-C. Trainor L. Gordon R. (2021). Rhythm and timing as vulnerabilities in neurodevelopmental disorders. *Philos. Trans. R. Soc. B Biol. Sci.* 376:20200327. 10.1098/rstb.2020.0327 34420385 PMC8380970

[B120] Lerdahl F. Jackendoff R. S. (1996). *A generative theory of tonal music, reissue, with a new preface.* Cambridge, MA: MIT Press.

[B121] Lewis J. W. Wightman F. L. Brefczynski J. A. Phinney R. E. Binder J. R. DeYoe E. A. (2004). Human brain regions involved in recognizing environmental sounds. *Cereb. Cortex* 14 1008–1021. 10.1093/cercor/bhh061 15166097

[B122] Loehr J. D. Palmer C. (2011). Temporal coordination between performing musicians. *Q. J. Exp. Psychol.* 64 2153–2167. 10.1080/17470218.2011.603427 21929475

[B123] Loehr J. D. Large E. W. Palmer C. (2011). Temporal coordination and adaptation to rate change in music performance. *J. Exp. Psychol. Hum. Percept. Perform.* 37 1292–1309. 10.1037/a0023102 21553990

[B124] London J. (2012). *Hearing in time: psychological aspects of musical meter.* Oxford: Oxford University Press.

[B125] Malerba P. Kopell N. (2013). Phase resetting reduces theta–gamma rhythmic interaction to a one-dimensional map. *J. Math. Biol.* 66 1361–1386. 10.1007/s00285-012-0534-9 22526842

[B126] Marder E. Bucher D. (2001). Central pattern generators and the control of rhythmic movements. *Curr. Biol.* 11 R986–R996. 10.1016/s0960-9822(01)00581-4 11728329

[B127] Marder E. Bucher D. Schulz D. J. Taylor A. L. (2005). Invertebrate central pattern generation moves along. *Curr. Biol.* 15 R685–R699. 10.1016/j.cub.2005.08.022 16139202

[B128] Mates J. (1994a). A model of synchronization of motor acts to a stimulus sequence: I. Timing and error corrections. *Biol. Cybern.* 70 463–473. 10.1007/BF00203239 8186306

[B129] Mates J. (1994b). A model of synchronization of motor acts to a stimulus sequence: II. Stability analysis, error estimation and simulations. *Biol. Cybern.* 70 475–484. 10.1007/bf00203240 8186307

[B130] McAngus Todd N. P. Brown G. J. (1996). Visualization of rhythm, time and metre. *Artif. Intell. Rev.* 10 253–273. 10.1007/BF00127682

[B131] McAuley J. D. (1995). *Perception of time as phase: toward an adaptive-oscillator model of rhythmic pattern processing* (Unpublished doctoral dissertation). Bloomington, IN: Indiana University.

[B132] Merchant H. Averbeck B. B. (2017). The computational and neural basis of rhythmic timing in medial premotor cortex. *J. Neurosci.* 37 4552–4564. 10.1523/jneurosci.0367-17.2017 28336572 PMC6596663

[B133] Merchant H. Honing H. (2014). Are non-human primates capable of rhythmic entrainment? evidence for the gradual audiomotor evolution hypothesis. *Front. Neurosci.* 7:274. 10.3389/fnins.2013.00274 24478618 PMC3894452

[B134] Merchant H. Grahn J. Trainor L. Rohrmeier M. Fitch W. T. (2015). Finding the beat: a neural perspective across humans and non-human primates. *Philos. Trans. R. Soc. B Biol. Sci.* 370:20140093. 10.1098/rstb.2014.0093 25646516 PMC4321134

[B135] Michon J. A. (1967). *Timing in temporal tracking.* Soesterberg: Institute for Perception RVO-TNO.

[B136] Mogan R. Fischer R. Bulbulia J. A. (2017). To be in synchrony or not? a meta-analysis of synchrony’s effects on behavior, perception, cognition and affect. *J. Exp. Soc. Psychol.* 72 13–20. 10.1016/j.jesp.2017.03.009

[B137] Morillon B. Schroeder C. E. (2015). Neuronal oscillations as a mechanistic substrate of auditory temporal prediction. *Ann. N.Y. Acad. Sci.* 1337 26–31. 10.1111/nyas.12629 25773613 PMC4363099

[B138] Morillon B. Schroeder C. E. Wyart V. (2014). Motor contributions to the temporal precision of auditory attention. *Nat. Commun.* 5 1–9. 10.1038/ncomms6255 25314898 PMC4199392

[B139] Näätänen R. Paavilainen P. Rinne T. Alho K. (2007). The mismatch negativity (MMN) in basic research of central auditory processing: a review. *Clin. Neurophysiol.* 118 2544–2590. 10.1016/j.clinph.2007.04.026 17931964

[B140] Nasuto S. J. Hayashi Y. (2019). “Anticipation in neurocybernetics,” in *Handbook of anticipation*, ed. Poli R. (Berlin: Springer), 249–284. 10.1007/978-3-319-91554-8_61

[B141] Nowicki L. Prinz W. Grosjean M. Repp B. H. Keller P. E. (2013). Mutual adaptive timing in interpersonal action coordination. *Psychomusicol. Music Mind Brain* 23 6–20. 10.1037/a0032039

[B142] Nozaradan S. Peretz I. Mouraux A. (2012). Selective neuronal entrainment to the beat and meter embedded in a musical rhythm. *J. Neurosci.* 32 17572–17581. 10.1523/jneurosci.3203-12.2012 23223281 PMC6621650

[B143] Nozaradan S. Peretz I. Missal M. Mouraux A. (2011). Tagging the neuronal entrainment to beat and meter. *J. Neurosci.* 31 10234–10240. 10.1523/jneurosci.0411-11.2011 21753000 PMC6623069

[B144] Palmer C. (1996). On the assignment of structure in music performance. *Music Perception* 14 23–56. 10.2307/40285708

[B145] Palmer C. Lidji P. Peretz I. (2014). Losing the beat: deficits in temporal coordination. *Philos. Trans. R. Soc. B Biol. Sci.* 369:20130405. 10.1098/rstb.2013.0405 25385783 PMC4240972

[B146] Patel A. D. Iversen J. R. (2014). The evolutionary neuroscience of musical beat perception: the action simulation for auditory prediction (ASAP) hypothesis. *Front. Syst. Neurosci.* 8:57. 10.3389/fnsys.2014.00057 24860439 PMC4026735

[B147] Pazdera J. K. Trainor L. J. (2022). Pitch-induced illusory percepts of time. *PsyArXiv* [Preprint]. 10.31234/osf.io/6fx87 PMC11865124 39658731

[B148] Phillips-Silver J. Trainor L. J. (2005). Feeling the beat: movement influences infant rhythm perception. *Science* 308:1430. 10.1126/science.1110922 15933193

[B149] Pikovsky A. Rosenblum M. Kurths J. (2001). *Synchronization: a universal concept in nonlinear sciences.* Cambridge: Cambridge University Press.

[B150] Pittman-Polletta B. R. Wang Y. Stanley D. A. Schroeder C. E. Whittington M. A. Kopell N. J. (2021). Differential contributions of synaptic and intrinsic inhibitory currents to speech segmentation via flexible phase-locking in neural oscillators. *PLoS Comput. Biol.* 17:e1008783. 10.1371/journal.pcbi.1008783 33852573 PMC8104450

[B151] Poeppel D. Assaneo M. F. (2020). Speech rhythms and their neural foundations. *Nat. Rev. Neurosci.* 21 322–334. 10.1038/s41583-020-0304-4 32376899

[B152] Polak R. (2010). Rhythmic feel as meter: non-isochronous beat subdivision in JEMBE music from Mali. *Music Theory Online* 16 1–26. 10.30535/mto.16.4.4

[B153] Rankin S. K. Large E. W. Fink P. W. (2009). Fractal tempo fluctuation and pulse prediction. *Music Percept.* 26 401–413. 10.1525/mp.2009.26.5.401 25190901 PMC4151502

[B154] Repp B. H. (2001a). Phase correction, phase resetting, and phase shifts after subliminal timing perturbations in sensorimotor synchronization. *J. Exp. Psychol. Hum. Percept. Perform.* 27 600–621. 10.1037/0096-1523.27.3.600 11424648

[B155] Repp B. H. (2001b). Processes underlying adaptation to tempo changes in sensorimotor synchronization. *Hum. Movement Sci.* 20 277–312. 10.1016/s0167-9457(01)00049-5 11517673

[B156] Repp B. H. (2002a). Automaticity and voluntary control of phase correction following event onset shifts in sensorimotor synchronization. *J. Exp. Psychol. Hum. Percept. Perform.* 28 410–430. 10.1037/0096-1523.28.2.410 11999863

[B157] Repp B. H. (2002b). Phase correction in sensorimotor synchronization: nonlinearities in voluntary and involuntary responses to perturbations. *Hum. Movement Sci.* 21 1–37. 10.1016/s0167-9457(02)00076-3 11983432

[B158] Repp B. H. (2003). Phase attraction in sensorimotor synchronization with auditory sequences: effects of single and periodic distractors on synchronization accuracy. *J. Exp. Psychol. Hum. Percept. Perform.* 29 290–309. 10.1037/0096-1523.29.2.290 12760616

[B159] Repp B. H. (2005). Sensorimotor synchronization: a review of the tapping literature. *Psychon. Bull. Rev.* 12 969–992. 10.3758/bf03206433 16615317

[B160] Repp B. H. (2008). Multiple temporal references in sensorimotor synchronization with metrical auditory sequences. *Psychol. Res.* 72 79–98. 10.1007/s00426-006-0067-1 16786353

[B161] Repp B. H. Bruttomesso M. (2009). A filled duration illusion in music: effects of metrical subdivision on the perception and production of beat tempo. *Adv. Cogn. Psychol.* 5 114–134. 10.2478/v10053-008-0071-7 20689669 PMC2916667

[B162] Repp B. H. Doggett R. (2007). Tapping to a very slow beat: a comparison of musicians and nonmusicians. *Music Percept.* 24 367–376. 10.1525/mp.2007.24.4.367

[B163] Repp B. H. Keller P. E. (2004). Adaptation to tempo changes in sensorimotor synchronization: effects of intention, attention, and awareness. *Q. J. Exp. Psychol. A.* 57 499–521. 10.1080/02724980343000369 15204138

[B164] Repp B. H. Su Y.-H. (2013). Sensorimotor synchronization: a review of recent research (2006–2012). *Psychon. Bull. Rev.* 20 403–452. 10.3758/s13423-012-0371-2 23397235

[B165] Righetti L. Buchli J. Ijspeert A. J. (2006). Dynamic Hebbian learning in adaptive frequency oscillators. *Phys. D Nonlinear Phenomena* 216 269–281. 10.1016/j.physd.2006.02.009

[B166] Rinzel J. Ermentrout G. B. (1998). “Analysis of neural excitability and oscillations,” in *Methods in neuronal modeling: from ions to networks*, eds Koch C. Segev I. (Cambridge, MA: MIT Press), 251–291.

[B167] Rohenkohl G. Cravo A. M. Wyart V. Nobre A. C. (2012). Temporal expectation improves the quality of sensory information. *J. Neurosci.* 32 8424–8428. 10.1523/jneurosci.0804-12.2012 22699922 PMC4235252

[B168] Rohenkohl G. Gould I. C. Pessoa J. Nobre A. C. (2014). Combining spatial and temporal expectations to improve visual perception. *J. Vis.* 14:8. 10.1167/14.4.8 24722562 PMC3983934

[B169] Roman I. R. Roman A. S. Kim J. C. Large E. W. (2023). Hebbian learning with elasticity explains how the spontaneous motor tempo affects music performance synchronization. *PLoS Comput. Biol.* (in press). 10.1101/2020.10.15.341610 PMC10281589 37285380

[B170] Roman I. R. Washburn A. Large E. W. Chafe C. Fujioka T. (2019). Delayed feedback embedded in perception-action coordination cycles results in anticipation behavior during synchronized rhythmic action: a dynamical systems approach. *PLoS Comput. Biol.* 15:e1007371. 10.1371/journal.pcbi.1007371 31671096 PMC6822724

[B171] Sadakata M. Desain P. Honing H. (2006). The Bayesian way to relate rhythm perception and production. *Music Percept.* 23 269–288. 10.1525/mp.2006.23.3.269

[B172] Savage P. E. Brown S. Sakai E. Currie T. E. (2015). Statistical universals reveal the structures and functions of human music. *Proc. Natl. Acad. Sci. U.S.A.* 112 8987–8992. 10.1073/pnas.1414495112 26124105 PMC4517223

[B173] Savage P. E. Loui P. Tarr B. Schachner A. Glowacki L. Mithen S. (2020). Music as a coevolved system for social bonding. *Behav. Brain Sci.* 44:e59. 10.1017/s0140525x20000333 32814608

[B174] Savinov M. Swigon D. Ermentrout B. (2021). Synchronization and locking in oscillators with flexible periods. *Chaos* 31:033143. 10.1063/5.0021836 33810738

[B175] Scheurich R. Pfordresher P. Q. Palmer C. (2020). Musical training enhances temporal adaptation of auditory-motor synchronization. *Exp. Brain Res.* 238 81–92. 10.1007/s00221-019-05692-y 31792555

[B176] Scheurich R. Zamm A. Palmer C. (2018). Tapping into rate flexibility: musical training facilitates synchronization around spontaneous production rates. *Front. Psychol.* 9:458. 10.3389/fpsyg.2018.00458 29681872 PMC5897499

[B177] Schöner G. (2008). “Dynamical systems approaches to cognition,” in *The Cambridge handbook of computational psychology*, ed. Sun R. (Cambridge: Cambridge University Press), 101–126. 10.1017/cbo9780511816772.007

[B178] Schroeder C. E. Lakatos P. (2009). Low-frequency neuronal oscillations as instruments of sensory selection. *Trends Neurosci.* 32 9–18. 10.1016/j.tins.2008.09.012 19012975 PMC2990947

[B179] Schroeder C. E. Wilson D. A. Radman T. Scharfman H. Lakatos P. (2010). Dynamics of active sensing and perceptual selection. *Curr. Opin. Neurobiol.* 20 172–176. 10.1016/j.conb.2010.02.010 20307966 PMC2963579

[B180] Schubotz R. I. (2007). Prediction of external events with our motor system: towards a new framework. *Trends Cogn. Sci.* 11 211–218. 10.1016/j.tics.2007.02.006 17383218

[B181] Senzai Y. Fernandez-Ruiz A. Buzsáki G. (2019). Layer-specific physiological features and interlaminar interactions in the primary visual cortex of the mouse. *Neuron* 101 500–513.e5. 10.1016/j.neuron.2018.12.009 30635232 PMC6367010

[B182] Sloboda J. A. (1983). The communication of musical metre in piano performance. *Q. J. Exp. Psychol.* 35 377–396. 10.1080/14640748308402140

[B183] Snyder J. S. Large E. W. (2005). Gamma-band activity reflects the metric structure of rhythmic tone sequences. *Cogn. Brain Rese.* 24 117–126. 10.1016/j.cogbrainres.2004.12.014 15922164

[B184] Stanley K. O. Clune J. Lehman J. Miikkulainen R. (2019). Designing neural networks through neuroevolution. *Nat. Mach. Intellig.* 1 24–35. 10.1038/s42256-018-0006-z

[B185] Stefanics G. Hangya B. Hernadi I. Winkler I. Lakatos P. Ulbert I. (2010). Phase entrainment of human delta oscillations can mediate the effects of expectation on reaction speed. *J. Neurosci.* 30 13578–13585. 10.1523/jneurosci.0703-10.2010 20943899 PMC4427664

[B186] Stephen D. G. Stepp N. Dixon J. A. Turvey M. T. (2008). Strong anticipation: sensitivity to long-range correlations in synchronization behavior. *Phys. A. Stat. Mech. Applic.* 387 5271–5278. 10.1016/j.physa.2008.05.015

[B187] Stepp N. Turvey M. T. (2010). On strong anticipation. *Cogn. Syst. Res.* 11 148–164. 10.1016/j.cogsys.2009.03.003 20191086 PMC2827858

[B188] Strogatz S. H. (2015). *Nonlinear dynamics and chaos: with applications to physics, biology, chemistry, and engineering.* Boca Raton, FL: CRC Press. 10.1201/9780429492563

[B189] Tal I. Large E. W. Rabinovitch E. Wei Y. Schroeder C. E. Poeppel D. (2017). Neural entrainment to the beat: the “missing-pulse” phenomenon. *J. Neurosci.* 37 6331–6341. 10.1523/jneurosci.2500-16.2017 28559379 PMC5490067

[B190] Teki S. Grube M. Griffiths T. D. (2012). A unified model of time perception accounts for duration-based and beat-based timing mechanisms. *Front. Integr. Neurosci.* 5:90. 10.3389/fnint.2011.00090 22319477 PMC3249611

[B191] Teki S. Grube M. Kumar S. Griffiths T. D. (2011). Distinct neural substrates of duration-based and beat-based auditory timing. *J. Neurosci.* 31 3805–3812. 10.1523/jneurosci.5561-10.2011 21389235 PMC3074096

[B192] Thaut M. H. Tian B. Azimi-Sadjadi M. R. (1998). Rhythmic finger tapping to cosine-wave modulated metronome sequences: evidence of subliminal entrainment. *Hum. Movement Sci.* 17 839–863. 10.1016/s0167-9457(98)00031-1

[B193] Tichko P. Large E. W. (2019). Modeling infants’ perceptual narrowing to musical rhythms: neural oscillation and Hebbian plasticity. *Ann. N.Y. Acad. Sci.* 1453 125–139. 10.1111/nyas.14050 31021447

[B194] Tichko P. Kim J. C. Large E. W. (2021). Bouncing the network: a dynamical systems model of auditory–vestibular interactions underlying infants’ perception of musical rhythm. *Dev. Sci.* 24:e13103. 10.1111/desc.13103 33570778

[B195] Tichko P. Kim J. C. Large E. W. (2022). A dynamical, radically embodied, and ecological theory of rhythm development. *Front. Psychol.* 13:653696. 10.3389/fpsyg.2022.653696 35282203 PMC8907845

[B196] Todd N. (1985). A model of expressive timing in tonal music. *Music Percept.* 3 33–57. 10.2307/40285321

[B197] Trainor L. J. Marsh-Rollo S. (2019). “Rhythm, meter, and timing: the heartbeat of musical development,” in *The Oxford handbook of music and the brain*, eds Thaut M. H. Hodges D. A. (Oxford: Oxford University Press), 592–622.

[B198] Treisman M. (1963). Temporal discrimination and the indifference interval: implications for a model of the “internal clock”. *Psychol. Monogr. Gen. Appl.* 77 1–31. 10.1037/h0093864 5877542

[B199] Valdesolo P. Ouyang J. DeSteno D. (2010). The rhythm of joint action: synchrony promotes cooperative ability. *J. Exp. Soc. Psychol.* 46 693–695. 10.1016/j.jesp.2010.03.004

[B200] van der Steen M. C. Keller P. E. (2013). The ADaptation and Anticipation Model (ADAM) of sensorimotor synchronization. *Front. Hum. Neurosci.* 7:253. 10.3389/fnhum.2013.00253 23772211 PMC3677131

[B201] Vorberg D. Wing A. (1996). Modeling variability and dependence in timing. *Handb. Percept. Action* 2 181–262.

[B202] Vuust P. Witek M. A. G. (2014). Rhythmic complexity and predictive coding: a novel approach to modeling rhythm and meter perception in music. *Front. Psychol.* 5:1111. 10.3389/fpsyg.2014.01111 25324813 PMC4181238

[B203] Vuust P. Dietz M. J. Witek M. Kringelbach M. L. (2018). Now you hear it: a predictive coding model for understanding rhythmic incongruity. *Ann. N.Y. Acad. Sci.* 1423 19–29. 10.1111/nyas.13622 29683495

[B204] Wang X.-J. (2010). Neurophysiological and computational principles of cortical rhythms in cognition. *Physiol. Rev.* 90 1195–1268. 10.1152/physrev.00035.2008 20664082 PMC2923921

[B205] Wei Y. Hancock R. Mozeiko J. Large E. W. (2022). The relationship between entrainment dynamics and reading fluency assessed by sensorimotor perturbation. *Exp. Brain Res.* 240 1775–1790. 10.1007/s00221-022-06369-9 35507069

[B206] Wilson H. R. Cowan J. D. (1973). A mathematical theory of the functional dynamics of cortical and thalamic nervous tissue. *Kybernetik* 13 55–80. 10.1007/bf00288786 4767470

[B207] Wing A. M. Kristofferson A. B. (1973). The timing of interresponse intervals. *Percept. Psychophys.* 13 455–460. 10.3758/bf03205802

[B208] Winkler I. Háden G. P. Ladinig O. Sziller I. Honing H. (2009). Newborn infants detect the beat in music. *Proc. Natl. Acad. Sci. U.S.A.* 106 2468–2471. 10.1073/pnas.0809035106 19171894 PMC2631079

[B209] Yuste R. MacLean J. N. Smith J. Lansner A. (2005). The cortex as a central pattern generator. *Nat. Rev. Neurosci.* 6 477–483. 10.1038/nrn1686 15928717

[B210] Zamm A. Wang Y. Palmer C. (2018). Musicians’ natural frequencies of performance display optimal temporal stability. *J. Biol. Rhythms* 33 432–440. 10.1177/0748730418783651 29940801

[B211] Zanto T. P. Large E. W. Fuchs A. Kelso J. A. S. (2005). Gamma-band responses to perturbed auditory sequences: evidence for synchronization of perceptual processes. *Music Percept.* 22 531–547. 10.1525/mp.2005.22.3.531

[B212] Zemlianova K. Bose A. Rinzel J. (2022). A biophysical counting mechanism for keeping time. *Biol. Cybernet.* 116 205–218. 10.1007/s00422-021-00915-4 35031845

[B213] Zhou S. Masmanidis S. C. Buonomano D. V. (2020). Neural sequences as an optimal dynamical regime for the readout of time. *Neuron* 108 651–658.e5. 10.1016/j.neuron.2020.08.020 32946745 PMC7825362

